# Factors affecting the choice of delivery place in a rural area in Laos: A qualitative analysis

**DOI:** 10.1371/journal.pone.0255193

**Published:** 2021-08-02

**Authors:** Yoshiko Kawaguchi, Ahmad M. Sayed, Alliya Shafi, Sengchanh Kounnavong, Tiengkham Pongvongsa, Angkhana Lasaphonh, Khamsamay Xaylovong, Miho Sato, Mitsuaki Matsui, Atsuko Imoto, Nguyen Tien Huy, Kazuhiko Moji

**Affiliations:** 1 School of Tropical Medicine and Global Health, Nagasaki University, Nagasaki, Japan; 2 Department of Organic Chemistry, College of Pharmacy, Al-Azhar University, Cairo, Egypt; 3 Online Research Club (https://www.onlineresearchclub.org/), Nagasaki, Japan; 4 School of Medicine, American University of Caribbean, Sint Maarten; 5 Laos Tropical and Pubic Health Institute, Vientiane, Lao PDR; 6 Savannakhet Provincial Health Department, Savannnakhet, Lao PDR; Shahjalal University of Science and Technology, BANGLADESH

## Abstract

**Background:**

Home delivery (HD) without skilled birth attendants (SBAs) are considered crucial risk factors increasing maternal and child mortality rates in Loa PDR. While a few studies in the literature discuss the choice of delivery in remote areas of minority ethnic groups; our work aims to identify factors that indicated their delivery place, at home or in the health facilities.

**Methods:**

A community-based qualitative study was conducted between February and March 2020. Three types of interviews were implemented, In-depth interviews with 16 women of eight rural villages who delivered in the last 12 months in Xepon District, Savannakhet Province, Lao PDR. Also, three focus group discussions (FGDs) with nine HCPs and key-informant interviews of ten VHVs were managed. Factors affecting the choice of the delivery place were categorized according to the social-ecological model.

**Results:**

Our sample included five Tri women and two Mangkong women in the HD group, while the FD group included three Tri women, two Mangkong women, one Phoutai woman, two Laolung women and one Vietnamese. Our investigation inside the targeted minority showed that both positive perceptions of home delivery (HD) and low-risk perception minorities were the main reasons for the choice of HD, on the individual level. On the other hand, fear of complication, the experience of stillbirth, and prolonged labour pain during HD were reasons for facility-based delivery (FD). Notably, the women in our minority reported no link between their preference and their language, while the HCPs dated the low knowledge to the language barrier. On the interpersonal level, the FD women had better communication with their families, and better preparation for delivery compared to the HD group. The FD family prepared cash and transportation using their social network. At the community level, the trend of the delivery place had shifted from HD to FD. Improved accessibility and increased knowledge through community health education were the factors of the trend. At the societal (national policy) level, the free delivery policy and limitation of HCPs’ assisted childbirth only in health facilities were the factors of increasing FD, while the absence of other incentives like transportation and food allowance was the factor of remaining of HD.

**Conclusions:**

Based on the main findings of this study, we urge the enhancement of family communication on birth preparedness and birthplace. Furthermore, our findings support the need to educate mothers, especially those of younger ages, about their best options regarding the place of delivery. We propose implementing secondary services of HD to minimize the emergency risks of HD. We encourage local authorities to be aware of the medical needs of the community especially those of pregnant females and their right for a free delivery policy.

## Background

Despite the progress made over recent decades to upgrade maternal health around the globe; yet maternal mortalities are still deemed a considerable portion of deaths [1,2]. The majority of these mortalities occur in developing countries that lack antenatal care systems [3]. While giving birth in healthcare institutions lowers delivery mortalities, the utilization of these facilities did not supersede. Hence, the reduction in mortalities not only depend on the spread of delivery institutions but also depends on other factors that lower the usage of those facilities. Those factors should be unsealed for better maternal healthcare.

Lao People’s Democratic Republic (Lao PDR) is a Southeast Asian country that has 49 official ethnic groups [4]. About two-thirds of the population belongs to Lao-Tai ethnolinguistic family whose first language is the Lao language, and the rest of them are from other families [5]. The proportion of ethnic minorities is higher in a remote area; for instance, in the Xepon district of Savannakhet Province where over 83% of people are not from the Lao-Tai family [6]. Regarding maternal health, Lao PDR still has a high maternal mortality rate (MMR) of 197/100,000 live birth, while the lifetime risk of maternal death is 1 in 150 in 2015 [7]. On the other hand, the home delivery (HD) rate is still high in Lao PDR; with 34.5% in national-wide, and 38.4% in Savannakhet Province in 2017 [6].

Individuals residing in remote areas are less likely to deliver at health care facilities. Kasaye et al. highlight that the absence of a health facility within a 30-minute distance of walking significantly increased HD. Additionally, financial status also affects the choice of delivery location [8]. Meanwhile, the government of Lao PDR launched free delivery and child care policy where pregnant women, women giving childbirth, postpartum women (42 days after delivery) and children under five years old can receive medical and non-medical benefits (transport cost, transfer cost and food allowance) [9]. In Lao PDR, unlike other regions where recent studies found a relation between the education level and the HD [10], the majority of the population are low-educated which masked any potential effect [11].

Starting from the childbirth experience, the perception, easiness, convenience, nearness of health facilities and the financial cost are considered causes of lack of institutional usage. For instance, in Xepon; the people’s perception of childbirth was negatively skewed to the facility-based delivery (FD) [12]. Smith et al. have declared that previous experience of complications and prolonged labour played a vital role in swaying the preference for FD [13]. Fear of complication and trust in the quality of the delivery process constituted other reasons [14]. Besides, the experience with the healthcare facilities, lack of privacy, improper behaviours of HCPs reversed the pointer to the HD [14]. In a study, the researchers reported that Lao PDR has a poor quality of care, bad attitude of HCPs, and lack of privacy, which affected the preference of the FD [15]. The ministry of health in Laos plans to increase the number of midwives and train them to improve the quality of care. Currently, trained midwives increased from 88 to 1784 in six years from 2010 to 2015. Yet, two-thirds of health centres (HCs) did not have a midwife in 2014 [9].

In the present study, we aimed to illustrate the factors that determined the choice of delivery place between HD and FD. We investigated factors like women who gave birth, the health care providers (HCPs), village health volunteers (VHVs and their role on the delivery-place determination in the targeted community. Our findings hail the limitations of the strategies developed inside Lao PDR to upgrade the current policies for the improvement of delivery services.

## Methodology

### Study area

**Fig 1:**
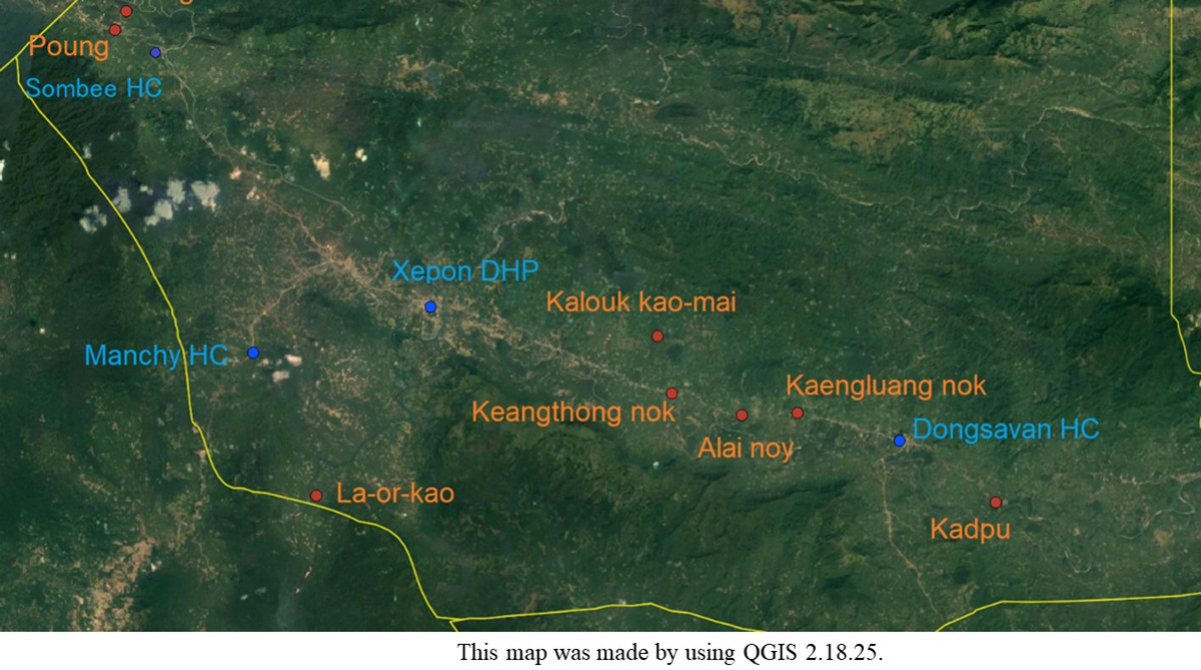
Map of the study site (Xepon district) with village names.

This study was conducted in villages of Xepon district which is in the northeast of Savannakhet Province, central of Lao PDR. Our inclusion criteria were to choose a village that: (1) their dwellers still using the HD. (2) to be about 5–20 km away from the closest health facility, Xepon District Hospital, and the healthcare centres. (3) to be covered in the Xepon HDSS. According to the pre-mentioned criteria and situation of COVID-19 pandemic; we have selected Vangmorthoum, Puong, La-or-kao, Keangthong nok, Kalouk kao-mai, Keangthong nok, Alai noy, Kaengluang nok and Kadpu. The total population of the eight villages is 2,236 in 2019 according to the Xepon district health office (DHO) (**Fig 1**).

### Study design and population

This community-based qualitative study was implemented in Xepon district, Savannakhet province between February and March 2020. The selection of women to volunteer in this study was performed with the support of two local research assistants from the Xepon District Hospital. The inclusion criteria for choosing our participants were to choose only women who: (1) had a child within 12 months, (2) a dweller of one of the eight villages, and (3) gave consent to participate. A total of 16 women who delivered a child within a year in the eight villages were selected with the support of Xepon District Hospital and the HCs. The interviews were conducted in each village. From each village, one woman who did HD and one woman who did FD were selected based on their availability and consensus to participate. Notably, one village of our selected 8 villages reported no HD within 12 months from the start of our investigation. In this village, we interviewed two women, who gave birth in the Xepon District Hospital and the HC, respectively.

### Data collection method

We collected data through three types of interviews: In-depth interviews (IDIs), focus group discussions (FGDs), key informant interviews (KIIs). Due to the low education level of the women inside our minorities; we could not use the surveys to collect data. However, the IDIs were conducted by the principal author in the Lao language and recorded by an IC recorder, with the permission of our participants, to explain their experience and point of view [16]. FGDs and KIIs were conducted to explain the group concerns, experiences and point of views [17]. The VHVs, HCPs or husbands interpreted from women’s local languages into Lao, when a woman could not speak Lao. The detailed results of IDIs, FGDs and KIIs are supported in (**Supplementary A, B and C** in S1 File).

### Data analysis

The interviews were audio-recorded and transcribed into English by YK. Then, the accuracy of her translation was checked by three native Laos speakers: TP, AL and KX to validate the translation. Those translations that included characteristics and statements for each participant are attached (**Supplementary D** in S1 File). Then, we divided the participants into HD and FD groups, to analyze their data. The factors that determined the decision-making of the delivery place were extracted from those interviews. Then, those factors were categorized and analysed manually according to the socio-ecological model.

### Validity and reliability

The validity/reliability of this data collection process is enhanced by the different sources used to extract data. Our sample size is representative of the rural community we are targeting, making this result to be generalized to a larger population.

### Ethical statement

Ethical approval was pre-obtained from both the National Research Ethics Committee for Health Research of Ministry of Health, Lao PDR and the Research Ethics Committee of School of Tropical Medicine and Global Health, Nagasaki University (**Supplementary E** in **S1 File**). Informed consent, in the Lao language explaining the research aims, was obtained from participants either by signing or thumbprint. Individuals’ data was anonymized by codes to ensure their privacy.

## Results

A total of sixteen individuals participated in the study; seven and nine participants had HD and FD, respectively. Our methodology was to choose from each village: an HD case and an FD case; however, in one village there was no HD case so we had to choose two FD cases. Among the FDs mothers; four mothers had a birth in Xepon District Hospital (DHP) while the rest delivered in the HC. The HD group and FD group had a mean age of 31.4 and 24.0 years with a range of 17–38 and 17–36, respectively. Animism is the principal religion with (62.5%). Nine participants received no education at all, while the rest received about) 0-9 (year of education. The majority of our participants (93.75%) were farmers, while only one participant was a daily labourer.

Regarding ethnicity, the women in the HD group were five out of Tri ethnicity and two women out of Mangkong ethnicity. However, the FD group included three Tri women, two Mangkong women, one Phoutai woman, two Laoolung women and one Vietnamese woman. Regarding the husband’s education, the husband in the HD group and FD group spent about 0.9 and 2.1 years in education, respectively (**Tables 1 & 2**).

**Table 1:** Characteristics data of our included participants.

		Home Delivery (n = 7)	Facility-based Delivery (n = 9)
**Age**	Average (year) (range)	31.4 (17–38)	24.0 (17–36)
**Ethnicity**	Tri	5	3
	Mangkong	2	2
	Phoutai	0	1
	Laolung	0	2
	Vietnamese	0	1
**Religion**	Buddhism	1	3
	Animism	6	4
	Christianity	0	2
**Occupation**	Farmer	6	9
	Day labourer	1	0
**Education period**	No education (number of people)	6	3
	Average (year) (range)	0.9 (0–6)	4.1 (0–8)
**Language (Lao language)**	Yes	3	6
	No	4	3
**Husband’s age**	Average (year) (range)	33.1 (19–40)	26.6 (19–37)
**Husband’s occupation**	Farmer	5	7
	Day labourer	2	2
**Husband’s education**	No education (number of people)	3	2
	Average (year) (range)	2.1 (0–7)	6.0 (0–13)
**Husband’s language (Lao language**)	Yes	4	7
	No	3	2
**Number of people (/household)**	Average (range)	7.1 (6–10)	6.9 (4–9)
**Property (transportation)**	Motorbike (only)	3	5
	Motorbike and tractor	2	2
	Nothing	2 2	2
**Marriage status**	Marriage	4	8
	Divorce	0	1
	Remarriage	3	0
Marriage age *first marriage	Average (year) (range)	17.6 (15–23)	18.0 (15–27)
**Age of first birth**	Average (year) (range)	18.7 (15–24)	18.9 (15–28)
**Number of births**	Average (times) (range)	4.8 (1–8)	3.1 (1–9)

**Table 2:** Detailed data of the included participants.

Village	Age	Delivery place	Ethnicity	Occupation	Education priod	Speaking the Lao language	Number of childbirths	ANC visits (times)	VHVs’ visits (times)
**1**	28	DHP	Laolung	Farmer	6	Yes	1	8	0
	22	HC	Phoutai	Farmer	5	Yes	4	5	2
**2**	20	DHP	Vietnamese	Farmer	7	No	2	1	0
	34	Home	Tri	Farmer	0	No	5	0	3
**3**	28	HC	Mangkong	Farmer	0	Yes	5	2	0
	38	Home	Mangkong	Farmer	0	Yes	6	2	5
**4**	17	HC	Mangkong	Farmer	6	Yes	1	5	4
	36	Home	Mangkong	Farmer	0	No	8	5	5
**5**	20	HC	Tri	Farmer	0	No	1	0	0
	37	Home	Tri	Day labourer	0	No	6	0	5
**6**	36	DHP	Tri	Farmer	0	No	9	4	5
	33	Home	Tri	Farmer	0	No	5	0	4
**7**	23	HC	Tri	Farmer	8	Yes	3	4	2
	25	Home	Tri	Farmer	0	Yes	3	2	2
**8**	22	DHP	Laolung	Farmer	5	Yes	2	5	4
	17	Home	Tri	Farmer	6	Yes	1	1	0

### Theoretical foundation and findings

**Fig 2:**
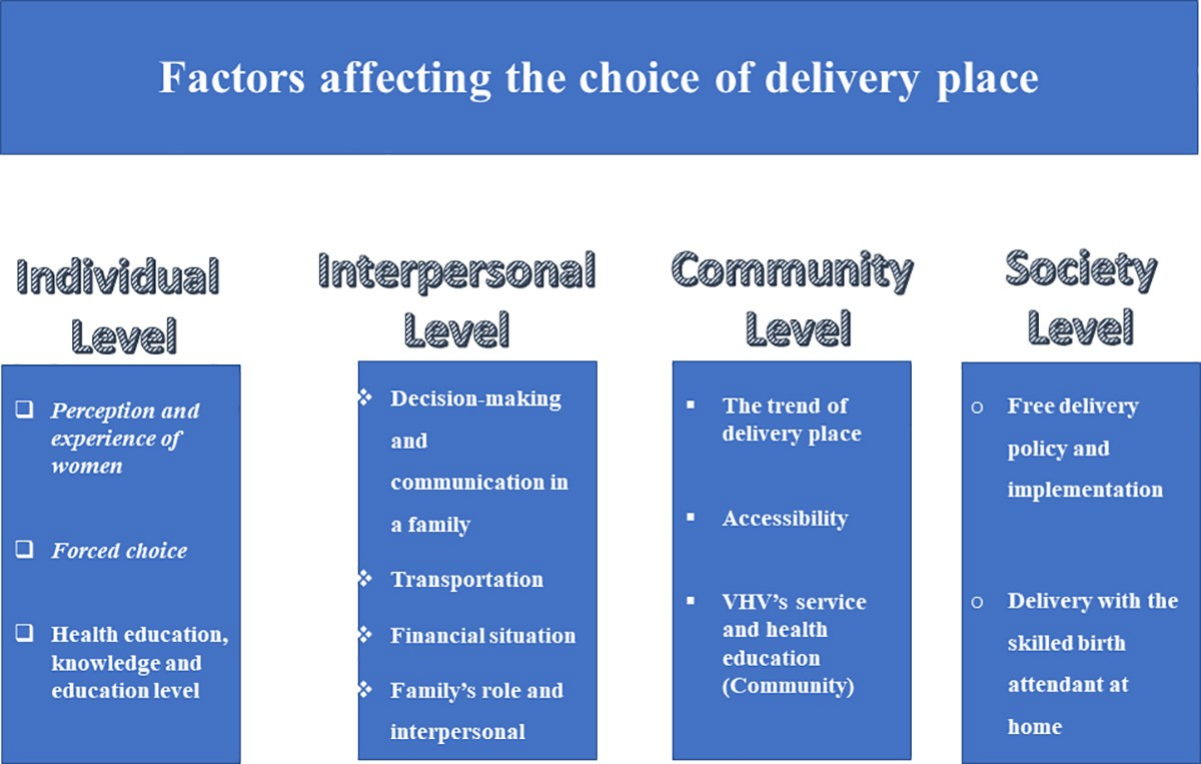
Summary of the factors affecting the choice of delivery place in a rural area in Laos.

In this study, we followed the social-ecological model of health [18] that categorized the factors determining the delivery place decision-making into 1) individual level, 2) interpersonal level, 3) community level, and 4) society level (national policy). In **Fig 2**, we have summarized all the reported factors under each category.

### The individual level

#### Perception and experience of women

Women who experienced HD showed a positive perception towards it, through using words like ‘‘Kud sabaii’ (or ‘Sabaii’) [easy delivery]. Some factors like the short duration of labour, dearth of complication/abnormal symptoms and normal pain during delivery endorsed this positive perception. For instance, the following is a quote from a mother:

“Childbirth is ‘Kud sabaii’, so I haven’t ever thought of FD.” (38yrs/HD/multipara).

On the other hand, many FD women clarified similar perception for FD. They said FD was ‘Sabaii’ (convenient). Some factors like the existence of HCPs, the medical care, the hospital equipment and the available medicines in the health facility bolstered the positive perception.

“There are medical staffs to help me in the HC. There is only my husband to help me at home. Husband, he doesn’t know his wife [her condition].” (28yrs/FD/multipara).

Some of them reasoned the rush for FD by the fear of difficult delivery or complications which was the main reason for thrusting them to FD.

“I decided to go to the hospital because we were afraid of some accidents [complications] happened. So, I went to the hospital.” (20yrs/FD/multipara).

Likely, the FD women without HD experience showed negative thoughts regarding the HD; as they recognized HD as unsafe, unhealthy and limited towards any abrupt complications.

“It is not safe to give a childbirth at home.” (28yrs/FD/multipara).

Woman with experience of delivering in the Xepon district hospitals also claimed the professionality of the HCPs.

“HCPs took good care and did monitoring well when I felt labour pain” (22yrs/FD/multipara).

Even women delivered at home showed a positive impact of FD due to awareness campaigns and person-to-person feedback.

“I know friends with FD experience, she said it was good to give a childbirth in the DHP.” (36yrs/HD/multipara).

The only negative opinion about FD was raised by a shy young mother.

“I didn’t want to go FD because of my shyness. Women who can do FD are not shy.” (25yrs/HD/multipara).

Unfortunately, five women (four HD women and one FD woman) experienced child deaths after HD; one woman lost her child two days after HD. However, this history did not affect their opinion and they insisted on their previous experience regardless of the bad experience.

#### Forced choice

HD women linked the need to go to the hospital/HC by the existence of a complication.

“I have to go to the hospital if the bleeding cannot stop. I think like that. There was no case like bleeding (in the past). I experienced only ‘Kud sabaii’. Previously, we didn’t know, nobody explains and provided health education. But I understand that now.” (36yrs/HD/multipara).

For example, two FD women experienced HD in the past and they moved to the hospital/HC after two- or three-days suffering from labour pain at home without any delivery.

“I intended to give a childbirth at home. But I had felt pain for two days without delivery. So, I went to the HC.” (38yrs/FD/multipara)

#### Health education, knowledge and education level

Regarding medical awareness, no women reported postnatal care (PNC). Concurrently, both numbers of antenatal care (ANC) visits and VHVs’ visits were similar in 5/8 villages (**Table 3**). Notably, one FD woman received neither ANC nor VHVs’ visit. Most women said they received health education from HCPs and/or VHVs during the latest pregnancies. Two women reported a lack of health awareness about the delivery place even though with their contact with ANC or VHVs. Some women could not identify the irregular symptoms, but basically, HD women recorded the same degree of knowledge as the FD group. The HCPs are linking this low knowledge with the education level and the language barrier. Nevertheless, the women did not raise the language barrier as a direct reason for HD. We found that women, who only speak their local language, could use a familial member for translation into the Lao language,

“One person has a good husband. The husband studied and understood health education. Another one, the husband says ‘Yes, yes.’ during health education, and they do not come at the timing of childbirth. He says ‘I am busy to go somewhere.’” (HCP in Manchy HC)

**Table 3:** Data of our participants related to their latest childbirths.

		Home Delivery (n = 7)	Facility based Delivery (n = 9)
**Antenatal care (ANC) visit**	No	3	1
	1–3 times	3	2
	More than 4	1	6
**village health volunteers (VHV) visit**	No	1	4
	1–3 times	2	2
	More than 4	4	3
**Health education about the delivery place**	Yes (received)	6	9
	No	1	0
**Health education provider**	HCPs/VHVs/Other	4	6
	HCPs/VHVs	0	2
	VHVs/Other	1	1
	Husband/Parents-in-law	1	0
**‘Yu-fai’^1)^ duration (days)**	Average (range)	6.1 (3–11)	7.3 (3–12)
**‘Yu-fai’ place**	House (including kitchen)	4	9
	Small hut	2	0
	Small hut and house	1	0
**‘Yu-fai’ tool for fire**	Charcoal	0	2
	Wood	7	7
**Food restriction after delivery**	Yes	1	3
	No	6	6

^1)^ ‘Yu-fai’ is a traditional postpartum custom in Lao PDR. Women after delivery stay and take a rest near the fire.

### Interpersonal level

#### Decision-making and communication in a family

In the FD group, the interfamilial conversation during pregnancy was more active than in the HD group. The FD-women mainly concluded FD with their husbands.

“I and husband talked together that we would go to the hospital or the HC, or at home for our childbirth. My husband initiated the discussion. (…) I replied it was ‘Sabaii’ to give childbirth in the hospital.” (23yrs/FD/multipara).

“I talked with my family where I would go for my childbirth, and how we would go.” (22yrs/FD/multipara).

In some cases, parents or parents-in-law participated in the decision-making. Women and their families seemed to reach an agreement to do FD without dissent in each family.

“No, parents-in-law didn’t say anything about our decision to do FD. They said that it was ‘Sabaii’ to give childbirth in the HC.” (23yrs/FD/multipara)

Some of the HD women were the main decision-makers, as the HD families did not take the delivery place seriously. Therefore, they had an access to the FD; however, they chose the HD.

“My husband asked me whether to go to the hospital. I rejected it because of ‘Kud sabaii’[easy delivery].” (36yrs/HD/multipara).

“My husband said he would take me to the HC for a childbirth. I said I didn’t want to go.” (25yrs/HD/multipara).

Even one of our cases preferred to go for the FD, however, she went to the HD unintentionally. She had labour pain so they rushed to the HD.

“I tried to say to take me to the HC. But my husband didn’t understand. I was painful and tried to say. My husband said that I ate too much spicy food. (…) I felt angry. I felt angry with my husband. I hoped him to take me to the HC.” (17yrs/HD/primipara).

Only three young mothers depended on their decisions over their families.

“My mother-in-law said to me to go to the HC for a childbirth because of fear of difficult birth. (…) I didn’t feel anything against mother-in-law’s decision, just went.” (20yrs/FD/primipara).

#### Transportation

Inadequate proper means of transportation and the abrupt labour pain constituted the reasons for the HD preferability.

“There was no vehicle to go.” (33yrs/HD/multipara).

“I felt labour pain in the big house to sleep. I moved to the small hut on foot and gave a childbirth (she explained a short duration of labour).” (25yrs/HD/multipara).

Our findings showed that there is no significant difference between the two groups regarding the means of transportation. Directing to the health facility was done through owned motorbikes or hired means of transportation.

“We talked to look for a car, to go by car. I talked together.” (22yrs/FD/multipara).

#### Financial situation

Both interviewed mothers and the VHVs agreed that the finance may be considered a reason directing towards a delivery place, but it is not the only one. Notably, about 42% of our sample dated the preference for HD to poverty. However, one HCP said it was a good excuse not to do FD. The VHVs reasoned this to the absence of cash or the will to pay.

“HD woman said she had no money and did not go to the hospital; she would go if she had money. It is easy to say.” (HCP in DHP).

“They would prefer to use the money for something else because they have no complication during delivery. It is easy, a baby is born.” (VHV).

While the authorities in Lao PDR implement the free delivery policy, the policy is not notorious for women. In addition to the added cost for FD like gasoline fee, food and unofficial payment to HCPs as a token of appreciation. Therefore, they tended to take the cost as a burden.

FD cost the families to pay for gasoline, food, water and baby goods like clothes. Some women reported an unofficial payment to HCPs as a token of appreciation. On the other hand, HCPs and VHVs said that women are not obliged to pay for HCPs any additional payment.

“And ‘Kha mu’ [unofficial payment to HCPs] they want to give 10,000 or 20,000 kip? It depends on them. It is possible not to pay them if they don’t have money.” (VHV).

Cash preparation during pregnancy was mentioned only in the FD group. One young FD woman did not know the policy of free delivery or the details of cost because her husband covered it. Normally, families get money from hunting, gathering, selling livestock, day labour and saving.

“I prepared the mother and child book, diaper, cloth for holding a baby, Lao skirt [a traditional skirt] and money.” (22yrs/FD/multipara).

One multipara received finance and transportation support from her family because of a shortage of money. In her case, she did not arrange this support in advance because she did not plan FD.

“I borrowed money from my brother-in-law [the husband of her old sister]. I did not have, and my husband did not have money.” (36yrs/FD/multipara).

#### Family’s role and interpersonal relationship

Preparation for delivery was common in the FD group more than in the HD group. In contrast, three HD families constructed a small hut for HD between six to eight months of pregnancy.

“I told my husband when it was near childbirth. My husband found a small hut as a place to childbirth.” [Her husband constructed the small hut around six months of pregnancy.] (38yrs/HD/multipara).

One male VHV mentioned it was one of the husband’s role to stay with their wives in the last month of pregnancy.

“A wife is pregnant and she will give a childbirth this month, her husband doesn’t go anywhere and have to stay with his wife to take care of his wife, go together anywhere she wants to go.” (VHV).

Relatives and friends with experience of FD played the role of giving information to HD women.

“I heard. My relatives said the experience of FD before.” (34yrs/HD/multipara).

A male VHV also mentioned villagers shared their experience with others and there was a possibility of persuasion to change their behaviour.

“Women who gave a childbirth at a health facility before explain to their friend when they gave a childbirth and felt happy.” (VHV).

Factually, there are cases that villagers positively affect HD by agreeing with HD.

“Relatives said that HD was ‘Kud sabaii’. Not afraid.” (36yrs/HD/multipara).

Notably, the HD husbands supported their wives through the delivery process including other members of their families. The support extended to include direct care as receiving a baby, cutting an umbilical cord with a razor or wood, and placenta delivery.

“My husband holds my body. There were me and a friend inside, my husband. And they received me. I did strain and gave childbirth. Next, cord-cutting, I cut it by myself, and a placenta came out by myself. Only mother-in-law and my friend wiped a baby and hold a baby by cloth.” (34yrs/HD/multipara).

On the other hand, the FD husbands’ missions were to prepare the cash and transportation for their wives to the hospital or HCs. Mother-in-law and female relatives accompanied them.

.“My husband took me to the HC. I went with my husband and old sister, two people.” (28yrs/FD/multipara)

The role of relatives in health facilities was to psychologically support mothers. They had no rule regarding health care such as taking care of the new-born. For instance, a husband entered a delivery room and encouraged his wife.

“My husband and parents and relatives did not help anything. They went and just observed.” (28yrs/FD/primipara).

### Community-level

#### The trend of delivery place

In two remote villages with difficult means of transportation, HD is still common. The VHVs accepted the HD and recommended the shift to FD in case of delivery linked complications.

“They have to go to the HC if there are symptoms of complications. If there is nothing, not painful, common pain, they just give a birth at home.” (VHV).

“Strong pain makes a husband take his wife to the hospital. He is afraid that a problem happens. Because…”(VHV).

On the other hand, the shift of delivery place from home to health facilities became a recent trend in Xepon district including the targeted villages of this study.

“The smaller number of women do HD than before.” (VHV).

#### Accessibility

One hour and a half to reach the HC, or to use the hard off-road raised the fear of women giving birth on the road or facing troubles. The distance and inaccessibility of women to reach the centres skipped them to the HD.

“Uh. The road is difficult. If the road is available, then it’s easier to go FD.” (VHV).

“We want to give a childbirth in the HC, but afraid to give a childbirth on the way.” (VHV).

The other VHVs and HCPs mentioned the construction of the 9E road (main road of Xepon district which connects the centre of Savannakhet Province to Vietnam) contributed to the hike in the number of FD.

“In 2010–2012, the road [9E road] was completely opened and it became convenient.” (HCP in DHP).

On the other hand, some VHVs did not agree that improving accessibility like the new road or building new HCs directly affect the delivery place. They mentioned other reasons to change people’s behaviour.

“They can go to the HC, but FD hasn’t been all yet. Around this (latest) three years, they come to the HC for childbirths. There are still people with HD.” (HCP).

#### VHV’s service and health education (community)

The VHVs’ mission is to raise medical awareness for pregnant women and their families; three women with no ANC visits received their visits from the volunteers.

“Because they understand to go to the HC. Delivery in an HC is safe. There is no right thing at home. If they use the incorrect thing, it is not healthy, pain and fever, mother and child. Tool for delivery at home is not like ones in an HC, not clean. We are afraid of becoming diseases. Many people understand that and go to HC.” (VHV).

VHVs reported that awareness campaigns contributed to improving their knowledge about the benefit of FD, risk of HD and free delivery policy. Therefore, more people choose FD than before.

### Societal level

#### Free delivery policy and implementation

The free delivery policy implemented in the region granted the women free service; however, they did not receive non-medical support like transportation fees. Besides, some women reported their ignorance regarding this policy.

#### Delivery with the skilled birth attendant at home

Limited resources hindered the HCPs to introduce the medical services at home; so, women need to go to the facilities to receive the service from the skilled birth attendant.

“They come if they have a car. In the case that a placenta does not come out after HD, a family comes to an HC. But the staff does a night shift alone (she cannot go to a village), so we ask them to take a mother and child here.” (HCP).

## Discussion

This study is formed as a community-based qualitative study to be able to figure out the answers to its aim. As we intended to extract data about the social behaviour of the minorities living in those rural villages in Lao PDR; we had to use qualitative research methods. The reason is that the quantitative methods are known for their limitations in describing the social experiences [19]. Besides, this research is investigating the behaviour of minorities that have no many published reports about them; therefore the qualitative approach was selected to give in-depth information about the problem [20]. From the quantitative methods; the community-based was selected to involve the minorities community to draw their social preferences and reasoning it form their exact point of view [21]. Using the community-based model we had *surveys*, *interviews*, *focus groups*, *observations or the literature*. *Coming to literature*, *the data published are rare*. *Also*, *choosing the surveys was not a good choice due to the low education level and the misunderstanding that may happen*. *Therefore*, *we used all that we can to extract the responses*.

Our findings suggested that family communication influenced the delivery place and the preparation for childbirth. Unlike some studies [12,22], we found that our participants had more autonomy among the Lao PDR minorities to choose their delivery place. We can date this freedom to the surge of public awareness and the implementation of new policies which have empowered pregnant women. On the other hand, we found that when the communication inside the family abates, the HD upsurges. This is supported by Kifle et al. who stated that the couple is likely to choose the FD when they choose together more than if one of them decides alone [23]. Moreover, Kabakyenga et al found that the discussion with a third party, like the HCPs, boosted the possibility of FD [24]. Overall, interfamilial communication alongside medical awareness can scale down HD.

Besides, the pre-preparation for birth was more familiar in the FD group. Our finding is coming in accordance with a study that stated that women with good preparedness for birth were skewed to the FD [25]. As well, Stiefane et al. have figured a rope between the institutional delivery and the rehearsal for birth [26], which is backing our finding. In Lao PDR, the current “mother-and-child book” urges the people to create a plan about the delivery place, companion(s), transportation and the financial issues for delivery. Accordingly, this step should boost the direction of new mothers towards the FD. Currently, there is a responsibility on the HCPs with the VHVs to aware the pregnant women including their families to establish their plan and follow the guidelines mentioned in the “mother-and-child-book”.

Surprisingly, we reported that the crucial factor supporting HD is the inherited perception of upcoming mothers from their ancestors. In accordance, previous studies found women, with previous experience with the HD, continued to implement it [8,12]. Another study found that the low risk in birth addressed women to acknowledge the HD [27]. Overall, the multiparas continued to conduct the HD even after they acknowledged the risks, and it is hard to convince them of the opposite. On the opposite, women who experienced FD continued this path. Hence, it is better to focus the effort on primiparas to reduce the total number of HD. Notably, the awareness should be introduced to primipara with their families, as the young mothers are likely to depend on their families in their decisions. In literature, young mothers tended to give birth to institutions [27].

On the other hand, our findings revealed that multiparas intend to repeat the experience which hails the importance of making it easy for them to get help if any complications happened during delivery. Issues like illness perception, finance, distance/transportation should be studied carefully to reduce the number of HDs [28]. Minimizing the risk of HD is not avoidable to reduce the mortality rates among mothers and babies. For instance, the HCPs and VHVs do have a responsibility to educate women and their families on the critical timing that they should switch to FD. Moreover, they should work with the community to ease transportations in such cases. Furthermore, the availability of skilled birth attendants inside the Lao PDR communities may solve this dilemma.

Our findings determined the absence of postnatal care for the mothers included in this study, which resulted in the absence of any assessment or preventive measures shared with the families. On the other hand, the literature is stating that postnatal care significantly reduced neonatal death [29]. The value of postnatal care is not only to promote FD but also to increase the care and reduce morbidities and mortalities. Therefore, the WHO inquire that the first postnatal care should be done on the first day after delivery [30].

Our findings found that women received the medical benefits of the free-delivery-policy and no one did receive the non-medical benefits (transport cost, transfer cost and food allowance) that have been included. Therefore, we think that to get the optimum benefit out of the free delivery policy; a wider and deeper awareness campaign should be performed to cover most pregnant women. The respondents mentioned the financial difficulty as an obstacle of FD, even with their knowledge about free delivery. Therefore, non-medical support constituted a supportive idea to promote FD. In a study in Nepal, incentive and free delivery programs could enhance the FD [31].

### Limitation of the study

The main limitation of this study is the small number of respondents. Therefore, these findings are not sufficient for generalization to the population. We intended to investigate more villages; but the pandemic Covid-19 hindered our progress. In the targeted villages, people speak the Lao language and/or their tongue which shaped a barrier for communication. There was a possibility that the opinions of women who cannot speak the Lao dialect were modified. Besides, there was a possibility of social-desirability bias. Besides, we could not stay enough time to observe the situation of the villages because of COVID-19. However, with the few limitations we mentioned, this paper is providing full detail regarding the remoted-area women to improve maternal care services.

### Conclusion

In this study, we have found that the inherited perception of HD and the low risks of the delivery process constituted the main factor that propagated the choice of HD. Meanwhile, prolonged labour pain with complications played a vital role in the designation of institutional birthing. We figured, also, that the hike in the interfamilial communication with the good preparation of the delivery process directed the mothers towards the FD. Advantageously, the birth trend is switched towards the FD because of the raised knowledge and improved accessibility. Our results suggest increasing the awareness of the non-medical benefits of the Lao PDR free delivery policy and to boost the number of skilled birth attendants. Finally, the spread of the FD and childbirth with SBAs are critical in reducing both morbidities and mortalities among mothers and babies. Besides, we urge the healthcare authorities to launch awareness campaigns to introduce the community in those rural places to their free delivery policies.

## Supporting information

S1 File Additional files. Supplementary A: Focus group discussions (FGDs) with health care providers (HCPs). Supplementary B: Key informant interviews (KIIs) to Village health volunteers (VHVs). Supplementary C: Questionnaire/interview guides used for the research. Supplementary D: Summary sheets of women’s characteristic and statements. Supplementary G: Ethical approvals.
(DOCX)
