# Supplementary material for: Factors affecting the choice of delivery place in a rural area in Laos: A qualitative analysis
Source: PLoS One. 2021 Aug 2;16(8):e0255193. doi: 10.1371/journal.pone.0255193 (PMC8328292; doi:10.1371/journal.pone.0255193)
Supplement: S1 File — Supplementary A: Focus group discussions (FGDs) with health care providers (HCPs). Supplementary B: Key informant interviews (KIIs) to Village health volunteers (VHVs). Supplementary C: Questionnaire/interview guides used for the research. Supplementary D: Summary sheets of women’s characteristic and statements. Supplementary G: Ethical approvals. (DOCX) [file pone.0255193.s001.docx]

**Factors affecting the choice of delivery place in a rural area in Laos: a qualitative analysis.**

*Running title: Choice of delivery place in a rural area in Laos*

Yoshiko Kawaguchi^1,#^, Ahmad M. Sayed^2,3, #^, Alliya Shafi^3,4^, Sengchanh Kounnavong^5^, Tiengkham Pongvongsa^6^, Angkhana Lasaphonh^5^, Khamsamay Xaylovong^1^, Miho Sato^1^, Mitsuaki Matsui^1^, Atsuko Imoto^1^, Nguyen Tien Huy^1,3,*^, Kazuhiko Moji^1,*^

**Authors details**

^1^School of Tropical Medicine and Global Health, Nagasaki University, Nagasaki, Japan. ^2^ Department of Organic Chemistry, College of Pharmacy, Al-Azhar University, Cairo 11884, Egypt. ^3^Online Research Club (https://www.onlineresearchclub.org/), Nagasaki, Japan. ^4^American University of Caribbean, School of Medicine, Sint Maarten. ^5^Laos Tropical and Pubic Health Institute, Vientiane, Lao PDR. ^6^Savannakhet Provincial Health Department, Savannnakhet, Lao PDR.

^#^Authors equally contributed the work.

*Correspondence to: Kazuhiko Moji, School of Tropical Medicine and Global Health, Nagasaki University, Nagasaki, Japan (E-Mail:  [[moji-k@nagasaki-u.ac.jp](mailto:moji-k@nagasaki-u.ac.jp)](mailto:%20yyoshino@nagasaki-u.ac.jp)). Or Nguyen Tien Huy, School of Tropical Medicine and Global Health, Nagasaki University, Japan. (E-Mail: tienhuy@nagasaki-u.ac.jp)

**Emails :**

YK: [kawaguchi.y201951@gmai.com](mailto:kawaguchi.y201951@gmai.com).

AMS: [a.sarhan932020@gmail.com](mailto:a.sarhan932020@gmail.com) (ORCID: 0000-0003-0244-3457).

AS: [alliyashafi@gmail.com](mailto:alliyashafi@gmail.com) (ORCID: [0000-0001-8575-807X](https://orcid.org/0000-0001-8575-807X)).

SK: [sengchanhkounnavong@hotmail.com](mailto:sengchanhkounnavong@hotmail.com).

TP: [tiengkhampvs@gmail.com](mailto:tiengkhampvs@gmail.com).

AL: [tockykpn@gmail.com](mailto:tockykpn@gmail.com).

KX: [khamsamay4477@gmail.com](mailto:khamsamay4477@gmail.com).

MS: [mihos@nagasaki-u.ac.jp](mailto:mihos@nagasaki-u.ac.jp) (ORCID: 0000-0002-5220-1715).

MM: [mmatsui@nagasaki-u.ac.jp](mailto:mmatsui@nagasaki-u.ac.jp) (ORCID: 0000-0003-4075-1266).

AI: [imoto@nagasaki-u.ac.jp](mailto:imoto@nagasaki-u.ac.jp) (ORCID: 0000-0003-2744-6160).

NTH: tienhuy@nagasaki-u.ac.jp (ORCID: 0000-0002-9543-9440)

KM.: [moji-k@nagasaki-u.ac.jp](mailto:moji-k@nagasaki-u.ac.jp) (ORCID: 0000-0002-7543-1249).

## Supplementary A: Focus group discussions (FGDs) with health care providers (HCPs)

**1. Research Methodology**

Three focus group discussions (FGDs) were conducted in Xepon district hospital (DHP), Dongsavan and Manchy health centres (HCs) in Xepon district, Savannakhet Province between February and March 2020. It aimed to understand factors that affect women to choice of delivery place and women’s situation in Xepon.

**1.1. Study setting**

There are one DHP and 14 HCs in the Xepon district with 250 small villages. DHP covers 51 villages with 15,970 people; Dongsavan HC covers 43 villages with 8,676 people, and Manchy HC covers 21 villages with 6,697 people. Dongsavan HC is located 25km east of DHP. Manchy HC is 13km west.

**1.2. Participants and recruitment methods**

Ten health care providers (HCPs) who work in obstetrics ward or mother and child department in Xepon district hospital and two HCs (Dongsavan, Manchy) were selected by the support of two local research assistants from the Xepon District Hospital.

**1.3. Data collection methods**

Three FGDs were conducted in each health facility; DHP, Dongsavan and Manchy HCs between Febrary and March, 2020. These FGDs were run by the main author in Lao language and the research assistants helped. They were recorded by an IC recorder with the permission of our participants.

**1.4. Data analysis**

All FGDs were audio-recorded and transcribed into English by YK and two local assistants. Lao assistants checked the mistakes and misunderstandings of the first transcription. Characteristics of health facilities and participants, and their statements were described.

**2. Results**

**2.1. Basic information of the three health facilities**

Table 4 shows information about each health facility. In addition, DHP can provide Comprehensive Emergency Obstetric Care (EmOC) functions; surgery and blood transfusion. HCPs in all three health facilities did not assist HD at a village. They explained it was difficult to provide childbirth care at home because of limited human resource. All HCPs who work in the obstetrics ward and/or mother and child department provided health education to women, their companions and citizens. DHP conducted 50 first PNCs four hours after delivery (before discharge) and ten-second PNCs between two weeks and a month after delivery (Table 4 and 5). Dongsavan HC provided 12 first PNC two hours after childbirth and no second PNC; the number of first PNC was smaller than the number of deliveries. Manchy HC had no first PNC and 13-second PNCs between seven and 12 days after delivery. The number of second PNC was bigger than delivery because some women gave childbirth at DHP and came to the HC for PNC. There was no doctor in these two HCs. All HCPs including the hygiene management staff provided mother and child health (MCH) service including delivery assistant.

Table 1 Health facility information

| Facility name | | Xepon DHP | Dongsavan HC | Manchy HC |
| --- | --- | --- | --- | --- |
| Number of MCH services  (/month) | ANC | 210 | 42 | 21 |
|  | Delivery | 50 | 15 | 7 |
|  | HD assist | 0 | 0 | 0 |
|  | PNC | (PNC1)50  (PNC2)10 | (PNC1)12  (PNC2)0 | (PNC1)0  (PNC2)13 |
| Number of HCPs | Doctor | 8 | 0 | 0 |
|  | Assistant doctor | 6 | 1 | 2 |
|  | Midwife | 6 | 1 | 1 |
|  | Nurse | 18 | 1 | 1 |
|  | Primary health provider | 1 | 0 | 0 |
|  | Hygiene management | 0 | 1 | 0 |
|  | Total | 39 | 4 | 4 |
| EmOC contents which a facility has | Parenteral antibiotics | + | + | + |
|  | Uterotonic drugs | + | + | + |
|  | Parenteral anticonvulsant | + | - | - |
|  | Manual removal of placenta | + | + | + |
|  | Removal of retained products | + | + | - |
|  | Basic neonatal resuscitation | + | + | + |

In these three health facilities, women can access free delivery, therefore, they do not pay for FD. There was no incentive for women. In HCs, they provide basic ANC; weight, height, length of fundus uterine / waist circumference, blood pressure, oedema and fetal heartbeat check. Ultrasounds, non-stress test, urine and blood test are available only in DHP.

Three health facilities use sterilized scissors and instruments by autoclaves or boiled water. Skin-to-skin (5-30mins) and first breastfeeding around 10mins after delivery or after cleaning.

Table 2 Health education in three health facilities

| Facility name | | Xepon DHP | Dongsavan HC | Manchy HC |
| --- | --- | --- | --- | --- |
| Health education during ANC / pregnancy | Contents | Diet  Check fetus/moving Abnormal signs (bleeding, amniotic fluid leakage, Fever) | Diet  To prevent hard work, alcohol consumption and smoke | Diet/nutrition  No food restriction Sanitation Birth preparation (goods) for HD and FD |
|  | Timing | When there are abnormal signs | when they have abnormal signs | Every pregnant woman/everyday |
|  | Targets | Pregnant woman Companions (husband, relatives) | Women husband relatives | pregnant woman husband mother-in-raw |
|  | Place | ANC room villages  (every three months | HC villages  (every there month) | HC every village  (every month) |
|  | Methods | Only individual (No group education in a hospital) | Individual  Group education in the village | Only individual (no group education) |
| Health education during delivery | Contents | To take a rest medicine intake Yu-fai not to do food restriction to give mother's milk PNC visit | Diet Yu-fai how to take care of the child | No food restriction How to do Yu-fai haemorrhage |
|  | Timing | When giving a childbirth | in the bed / before departure | after delivery |
|  | Targets | Parents husband relatives | Relatives  and patient | Relatives who accompany a woman |
|  | Place | Delivery room or  when a patient takes a rest after birth | Delivery room | Delivery room |
|  | Methods | Oral, individual | Oral/individually | oral / individually |
| PNC | Contents for Mothers  (What are checked.) | Breast condition uterine skin colour vaginal and abdominal condition | Uterine recover breast, vaginal check | vagina skin colour  interview everything like diet |
|  | Contents for Children  (What are checked.) | skin colour umbilical cord mothers' milk suction breathing condition | skin colour  umbilical cord Suction  Breastfeeding | skin colour  umbilical cord |
|  | Contents - Vaccination/child | Hep B | Hep B | BCG, (Polio) |

^1)^ In DHP, most of the HCPs work in all department when they are night shift. One doctor and two midwives are in charge of obstetrics. Four midwives, one nurse and one primary health provider are in charge of mother and child departments.

According to HCPs who joined FGDs, they provide ANC, vaccination and health education about pregnancy, delivery, child care, vaccination and contraceptive medicines when they visit each village. The freaquency of these outreach activities is different in each facilities, between every month and three months.

**2.2. Characteristics of participants**

Four HCPs in Xepon DHP, three HCPs in Dongsavan HC and two in Manchy HC participated in FGDs. Table 6 shows the characteristics of participating HCPs. They were all female. The average of experience as an HCP was 17.8 years ranged between four and 40. Their ethnicities were Phoutai and Laolung.

Table 3 Characteristics of HCPs who participated in FGDs

| Name of health facility | | DHP | Dongsavan  HC | Manchy HC |
| --- | --- | --- | --- | --- |
| Number of participants | | 4 | 3 | 2 |
| Age | Average (years old) | 41.5 | 37.3 | 40.5 |
|  | 20s | 2 | 0 | 0 |
|  | 30s | 0 | 2 | 1 |
|  | 40s | 0 | 1 | 0 |
|  | 50s | 2 | 0 | 1 |
| Speciality | Doctor | 1 | 0 | 0 |
|  | Assistant Doctor | 0 | 1 | 1 |
|  | MW | 1 | 1 | 0 |
|  | Nurse | 1 | 0 | 1 |
|  | Primary health provider | 1 | 0 | 0 |
|  | Hygiene management | 0 | 1 | 0 |
| Experience (year) | Average (range) | 21.0 (4-40) | 13.7 (9-21) | 17.5 (7-28) |
| Ethnicity | Phoutai | 3 | 3 | 0 |
|  | Laolung | 1 | 0 | 2 |

**2.3. HCPs’ perception of FD and HD**

FD is safe because there are HCPs, sterilized equipment and medicines to help delivery. They have confidence to provide good care including environmental control like warm temperature for the newborn. It is happy, *‘Sabaii’*(convenient) because of cleaness. HCPs believe all people hope HD and come if they have money, care and good road condition.

There are some differences between the DHP and HCs. First, it is human resource; There are doctors and a specialist in the DHP; HCPs in the DHP receive trainings in the province, therefore they have better knowledge than HCPs in HCs who have less opportunities of training. Second, there are more medicines for treatment and equipment like ultrasounds in the DHP. They can deal with more severe complications and the cesarian section. One of HC roles is to detect complications like transverse position and recommend delivery in DHP. Third, they can transfer a patient to Savannakhet provincial hospital if necessary. In HCs, they can accept the normal case because of these limitations. In normal delivery, it is same in the DHP and HCs. However, some women prefer the DHP for their childbirths because there are more HCPs with skill like experts and more equipments. Women are recommended to stay two days after delivery, but the majority of them go back home two hours after delivery.

HD is possible for women. However, compared to FD, HD is not safe because of less people to help women and complications like bleeding. There are more risks of infection, for instance, cutting off an umbilical cord by non-sterilized equipment like shaped wood.

**2.4. Factors affecting women’s choice of delivery place**

**Individual level:**

***Perception and experience of women***

One reason for HD is *‘Kud sabaii’*; it seemed related to short duration of labour, no complication or no abnormal symptom and right or normal pain during delivery. For instance, multiparas and quick delivery within one hour.

“Some of them feel labour pain in the field. While they get things and prepare to go to HC, she sudden gives childbirth at home. There are some cases that childbirths happen in the car on the way go to an HC.”

They recognize some women switch from FD to HD when they feel they can give a childbirth smoothly.

“In some villages, there are women who come to birth for a first baby here, in the HC. They do HD for second baby because it is far,”

Some of HCPs still accept HD when they consider village situations.

“We can say that they give a childbirth at home if it is easy to birth. They are in a remote area, on the mountain. The labour pain got strong 2 days, 2 nights and she came here.”

Multiparas tend to repeat HD when they give first childbirths at home. Primipalas do HD because their parents had done HD. Of course, some women come to a health facility every time. On the other hand, not all women give childbirth at the same place every time. There are several patterns.

“There are many types of women. One woman came for FD. One did not come. There was a woman with 4 times deliveries. She did not come to an HC every time. Another woman with 4times deliveries came to the hospital twice, and did not come twice.”

***Forced choice***

People come to a health facility if a complication happen or they feel it is difficult delivery; it takes a long time like more than six hours. HCPs are afraid the timing is too late to save their health.

“…… Father, in a village, she loses blood much, so you will take her to the HC. It would be dead before reaching.”

***Health education, knowledge, and education level***

They recommend ANC, FD and health education including the risk of maternal death, complication and mother and child deaths. They seem to provide health education in each health facilities and in each village during outreach activities. Repeating health education is importation to change people to do FD even though it takes time. The targets are pregnant women, husbands, parents, parents-in-law and relatives. A DHP doctor mentioned they need VHVs and HCs to provide health education. Men tend to participate in a village meeting compared to women.

“We have to rely on VHVs to provide health education for them.”

“We have to rely on HCs when they give vaccination for pregnant women and child in the village so they should provide more health education for them.”

There are some problems against their recommendation; there is no ANC visit because husbands do not take them; a woman comes to ANC, but do HD, and delay of first ANC visit happens. Even though their husbands take women to ANC and receive health education, people who do HD cannot understand health education because of no education and language barrier. Women with language barrier have been silent until HCPs talk in their language. Husbands can understand the Lao language more. Besides, the difference between HD woman and FD one is the difference in the husband’s understanding of health education.

“One person has a good husband. The husband studied and understood health education. Another one, the husband says ‘Yes, yes.’, and they don’t come at the timing of childbirth. He says I am busy to go (somewhere).”

**Interpersonal level:**

***Decision-making and communication in a family***

Decision-makers in a family have changed from a father-in-law to a couple (woman and her husband) In the past, a father-in-law decided all by himself. In the current, more couples mainly talk and decide their delivery place. In this case, a father-in-law, a mother-in-law and brothers talk with them and participate decision-making partly. In this family, women’s opinion is respected from other family members.

“In the past, a father-in-law decided to do HD. Nowadays, he joined couple’s decision-making and understand it well.”

“When a wife asks her husband to take her to the hospital during labour pain, he takes her without asking (to her/his parents).”

However, there are families that a husband and a mother-in-law still had more power of decision-making. A woman can not join dicision-making or she has less opportunity to show her opinion. HD sometimes happens because of family’s disagreement or non-cooperation even though a woman hopes FD. It is also related to no/less ANC visit and the delay of first ANC.

“Decision-makers are husband, father-in-law, mother-in-law. A patient wants to come for FD, but a husband doesn’t allow it, a mother-in-law doesn’t allow, so she doesn’t come.”

Especially a husband tends to be a final decision-maker. However, not all husbands are cooperative. HCPs hope their parents support a woman to reach a health facility if a husband is uncooperative.

“If a husband doesn’t accompany his wife, a mother or father should decide to rent a car to go to health facilities. Currently, the majority of people give childbirth in health facilities and hospital.”

***Family’s role and interpersonal relationship***

In each health facility, HCPs mainly provide care to women. Husbands have no or less role during FD. Their husbands, parents and sisters company women. Husband just bring a placenta back and bury it in the soil. Husbands do not need to touch blood like HD.

“In HD, a husband touches her blood, not smell good. The husband does not want to do.”

***Transportation***

No transportation is also one reason of HD. People do not like to ride a motorbike during delivery. They prefer to use a tractor or a car. HCPs recommend not to use a motorbike when they go back home after delivery because we are afraid of dizziness and other trouble.

***Financial situation***

HCPs raise no money as one of reasons that people do HD.

**Community level:**

***The trend of delivery place***

There was no FD in the HC in the past. In the current, the majority of them come to FD. The FD proportion has been increasing in this district. In DHP target area, there are only a few HDs recently. These happen because of the short duration of childbirth. If HD happens, they come to the DHP before or after placenta delivery to receive care and observation from HCPs. In one HC area, approximately 50% of women give a childbirth in the HC, 25% in the DHP, and 25% at home. Women who have a car near the HC prefer to go to the DHP for childbirth because it is not far by car. They think a bigger health facility is better because there are more HCPs.

***Accessibility***

Women do HD because of accessibility; for instance, hard or bad road condition, long distance and rain. Another case is the husbands’ busyness or no person to take her to a health facility.

“Because their villages are far from the HC. First, the road is difficult. Second, it is easy to give childbirth.”

“Like La-or-Kao village, they live near the river. They arrive at the shore [cross a river] and then take a vehicle to the HC.”

***Ethnicity and religion***

They have no clear idea the relationship between delivery place and ethnic group. In one health facility, they say Tri women tend to do HD, but they have shifted from HD to FD. They generally think the more women receive ANC, the more they do FD. On the other hand, there are some gaps between the ANC visit and FD. Some people do not come to ANC but come to give childbirth. They image it is related to what they believe even though they did not explain the details.

“They respected the religion of God. They don’t come to ANC but come for childbirth. To feel strong pain or anything else, I don’t know.”

**Societal level:**

***Free delivery policy and implementation***

In these health facilities, women can access free delivery service, therefore they do not need to pay for FD. But there was no incentive, so they have to prepare for transportation, food and other goods if they need.

***Delivery with a skilled birth attendant***

They assist childbirth only in health facilities because of limited human resource. Women need to do FD to realize childbirth with SBAs. They seemed to provide child vaccination, ANC, PNC and family planning (providing contraceptive methods) as outreach activity in each village.

## Supplementary B: Key informant interviews (KIIs) to Village health volunteers (VHVs)

**1. Research Methodology**

Key-informant interviews with ten VHVs were conducted in the villages where women lived in the Xepon district between February and March 2020.

**1.1. Study setting**

These seven villages are located in the Xepon district. These villages are about 5-20 km away from the close health facility.

**1.2. Participants** **and recruitment methods**

VHVs who do their activities during the research period were recruited based on the list with the support of the Xepon District Health Office (DHO). The interviews were conducted in each village. VHVs who gave consent on the day were interviewed in their villages. Their participations were voluntary.

**1.3. Data collection**

KIIs were conducted in each seven village between February and March, 2020. These KIIs were run by the main author in Lao language and the research assistants helped. They were recorded by an IC recorder with the permission of our participants.

**1.4. Data analysis**

All KIIs were audio-recorded and transcribed into English by YK and two local assistants. Lao assistants checked the mistakes and misunderstandings of the first transcription. The characteristics of VHVs, villages, and their statements were described.

**2. Results**

**2.1 Characteristics of VHVs**

Out of the ten VHVs, seven were male and the other three were female. They were all farmers. Table 7 shows the VHVs’ characteristics.

Table 4 Characteristics of VHVs

|  | | Male VHV | Female VHV |
| --- | --- | --- | --- |
| Age (years old) | Average (range) | 37.6 (25-56) | 29.3 (28-32) |
| Experience as VHV (year) | Average (range) | 12.46 (0.25-22) | 2 |
| Ethnicity | Tri | 3 | 1 |
|  | Mangkong | 2 | 2 |
|  | Phoutai | 2 | 0 |
| Education | No education (person) | 0 | 1 |
|  | Average (year) (range) | 6.6 (5-9) | 1.7 (0-3) |
| Number of VHVs’ children | Total | 29 | 5 |
|  | Average (range) | 4.1 (1-10) | 1.7 (1-2) |
| Children’s birthplace | Home (HD) | 18 | 3 |
|  | Health facility (FD) | 11 | 2 |
| The number of VHVs divided by children's birthplace | Only HD | 1 | 1 |
|  | Only FD | 3 | 1 |
|  | HD and FD | 3 | 1 |

The mean age of male VHVs was 37.6 years old. The mean of experience as VHV was 12.46 years. Five were the minority group people (three Tri and two Mangkong), the other two were Phoutai. The mean educational period was 6.6 years. Only one male VHV had the experience of child deaths five times.

The mean age of female VHVs was 29.3 years old ranged between 28 and 32. Their experience as VHV was two years because they were recruited when the JICA grass-root project started. They were all the minority group people (one Tri and two Mangkong). The mean educational period was 1.7 years. Female VHVs had no experience of child death.

**2.2 Information about villages**

Table 8 shows the basic information of these villages from VHVs. The total population is 2,029. The average time to reach the closest health facility is 32 mins ranged five and 60 by motorbike. The main occupation was farmer, there were some villages where people earn money as a day laborer. There are electricity and water supply (groundwater, no piped water) in all villages. Six villages have an only elementary school, the other one has no school. Many VHVs mentioned more children went to school and speak Laotian in the current. However, there are children who drop out after first to three years. Fewer reprocudutive people graduated from elementary school. The estimated literacy rate includes these children, therefore the literacy rate of reproductive age especially women is estimated lower than that.

Table 5 Basic information on villages (n=7)

| Population | Total | 2029 |
| --- | --- | --- |
|  | Average (range) | 290 (142-460) |
| Road accessibility | All season available (By car/bike) | 5 |
|  | All season available by bike  (Only dry season available by car) | 1 |
|  | Only dry season available | 1 |
| Public transportation | Bus and Songthaew (every day) | 3 |
|  | Nothing | 4 |
| Distance to the closest market(km) | Average (range) | 20 (8-34) |
| Year of electricity supply start | Average (range) | 2012 (2005-12) |
| Year of groundwater supply start | Average (range) | 2010 (2005-19) |
| Literacy rate (%) | Average (range) | 51 (30-85) |
| Women’s age of marriage | Average (range) | 17.0 (15.0-18.0) |
| Women’s age of first childbirth | Average (range) | 18.6 (15.5-21.0) |

**2.3. VHVs’ activities and their perception of FD and HD**

Female VHVs mainly provide health check-up, male VHVs record and report monthly, and both of them provide health education to pregnant women and their families every month, even though there are diffrences of their visits in each village. They recommend ANC visits, FD and PNC to them, and explain about the risk of HD, FD, free delivery, material preparation including gasoline and PNC. They actively recommend primiparas to go to DHP in the HC area. These targets are women, husbands and parents. Theier intervention to pregnant women starts from around three to six gestational months. Recommendation of PNC visit is included in theise health education, but less women go to PNC because they are healthy without abnormal sign after childbirth. They do not assist women during HD as trained birth attendant. Some of VHVs visit women after childbirth.

FD is better than HD because of ‘Sabaii’ (convenient), not difficult, cleanness, HCPs to help, many types of equipment and medicines. No villagers including VHVs say bad things about the health facility service. VHVs trust in HCPs including the situation that complication happened. Some VHVs remembered maternal and child deaths during HD. Therefore, they recognize FD is safe. In addition, women do not need to do anything like catching a baby, treat placenta and blood when HCPs help them. VHVs think The DHP has many doctors and equipment; in a HC, HCPs and equipment are limited, but there are many services. They are satisfied with FD both in the DHP and HCs.

Compared to FD, HD is unsafe. There is no person to help during HD, therefore it is impossible to deal with complication. However, more VHVs mention that HD is still many and acceptable than HCPs.

**2.4. Factors affecting women’s choice of delivery place**

**Individual level:**

***Perception and experience of women***

Many people hope for FD, but HD women have problems; the experience of *‘Kud sabaii’*, easy childbirth including short duration of labour especially multiparas and absent of person to take her. Women with easy childbirth experience are afraid of childbirth on the way to go to a health facility. HD women raised not to reach a health facility, laziness and experience of HD as a reason of HD. Compared to HD women, FD women percept HD is dangerous.

***Forced choice***

Women do HD if it is easy childbirth, and they go to a health facility if it takes time without childbirth.

*“They have to go if there are symptoms of complications. If there is nothing, not painful, common pain, they just give a birth at home.”*

Some women choose FD because they are afraid of a difficult delivery.

***Health education, knowledge, and education level***

VHVs recommend ANC visit and FD during their health education because the information is important. They feel difficulty to make people understand the contents and change their behavior. In some village, there is less custom of ANC visit; when women feel good without dangerous symptom, they do not want to go. There are both opinions about the relationship between ANC and FD. VHVs in three villages where the number of ANC visit is same between woman with HD and FD, have no idea or raise other reasons how they choose delivery place. They confirmed the gap between the reality (HD) and these couples’ agreement to FD during pregnancy.

**Interpersonal level:**

***Decision-making and communication in a family***

Decision-maker is depended on family, there are several cases. VHVs guess they decide delivery together. Some VHVs have no idea how people decide delivery place in a family.

“Everyone can decide, it equivalent to each person decided.”

The other VHV think the Main decision-maker is a husband, but he has to take her to the DHP if she hopes that in the current. Parents-in-law just recommend FD, but do not participate in the decision-making. A female VHV agreed that the main decision-maker was a husband. She expressed women had less power to decide FD.

“A wife decides, cannot decide. A husband doesn’t go together.”

In this village, mainly men can have driving technique, so women depend on men to go out by bike. Terefore, women feel men are main decision-makers of delivery place. In contrast, the male VHV sayed depended on a family.

***Transportation***

No beheicle in a family is often raised as reason of HD. Besides, many VHVs guess that Villagers can borrow a vehicle like a bike, car each other if they hope FD. In this case, families with pregnant women prepare gasoline itself or gasoline fee. Some families borrow gasoline fee if necessary. It is more difficulut to access to a health facility if a husband can not ride a motorbike.VHVs in the village along 9E road mentioned the road constraction make more families own vehicle. It is related to increase FD.

*“There are more vehicles. We go to the hospital. It is convenient now. (…) Every house has a car, motorbike, or tractor.”*

In most of the target villages, major transportation is a motorbike. Some people go to the HC by tractor. The other women use a car to access to a health facility. One female VHV felt she could not ride a motorbike during labour pain.

***Financial situation***

Poverty is one of reasons to do HD; or FD women have money. VHVs explain women and their family about the free delivery policy, but people need to pay for transportation and food. Women and their families do not come to a health facility if they can not prepare gasoline fee. In addition, HCPs mention that some families pay unofficial payment to HCPs called *‘Kha mu’*; it is no obligation.

The range of money to need for FD are different in each village; one VHV guess 10,000kip for a round-trip and the other cost are depended on a family; and the other VHV mention the cost of FD around 100,000-200,000kip. It is not easy to prepare but possible because it is money for saving a life. They prepare money or borrow many for gasoline and so on. Many villagers are former, so they collect cash by planting commodity crop like banana and cassava, and selling animals like wild pigs. In some villages, they work as dairy labourer for cash in Chinese, Vietnamise and Laotian compalies, for instance, collecting rubber liquid and heavy labour. In one village, the allowance of day labour is 40,000kip/day in a Chinese company, 50,000kip/day in a Vietnamese company. According to the research assistant, there is a possibility to find new labour with better conditions if a village head would negotiate with the Xepon district even though VHVs do not know the fact.

One VHV mentioned they have less willing to pay for FD because of no complication and easy childbirth.

“They would prefer to use the money for something else because giving birth has no complication. It is easy a baby is born.”

***Family’s role and interpersonal relationship***

According to VHVs, both HD and FD families build a small hut for childbirth and ‘Yu-fai’ (postnatal care). It is temporally one and dismantlement. However, the only family with HD prepared the small hut in this interview. The construction is done by father-in-law and/or husband. People prepare medicinal plants for both HD and FD. The roles of husbands are to take care of each other, not to leave his wife alone and observe her in the last month of pregnancy.

There is no person to help HD; a couple have to do it by themselves. Comepared to HD, one role of husbands is to take her to a health facility. He and mother-in-law commonly acompany a woman. One female VHV had the experience of once HD and once FD because there was no person to take her at first childbirth. It is the role of husband or family to borrow money if they do not have enough cash before childbirth.

Villagers share their own experience with FD positively to others. People recognized that it is ‘*Sabaii’* to give childbirth in the HC. The existence of peer with FD experience is also important.

“Some people who had experienced childbirths in a HC advised others that childbirth in HC was good”

*“Women who gave a childbirth at a health facility before explain to their friend when they give a childbirth and felt happy.”*

**Community level:**

***The trend of delivery place***

The trend of the delivery place has shifted from HD to FD; HD was common for their parents’ and old persons’ generation. In fact, there was no HD woman in one village within one year. The VHVs believe that primiparas should go to DHP because they have no experience of childbirth and are afraid of dangerous.

“Currently, they want to childbirth in HC or hospital when they feel childbirth whether it is easy or difficult.”

“The smaller number of women do HD than before.”

***Accessibility and transportation***

The shift of delivery place happened around 2010 or 2012. It was the timing that 9E road (main road in the district) was completely opened and it became convenient.

“The timing of shifting the delivery place was when 9E road (main road) was completely opened and it becomes convenient. The change also contributed to transportation to each family.”

The new HC opening contributes to make physical distance shorten. They shift from HD to FD started after the HC opening in 2005. But it took time around three to five years that more women do FD in the HC.

“They can go the HC, but it hasn’t been all yet. Around this (latest) 3 years, they come to the HC for childbirths. There are still people with HD.”

The other VHV in a village along the road emphasize the other factors as a reason of the shift.

“There is the improvement of accessibility like 9E road or HC opening. But they are not linked to a dramatic shift of delivery place. They mentioned other reasons to change people’s behavior.”

HD is still major especially in two target villages. One of their common points compared to the other villages is long distance to health facilities. For instance, one village people need one and a half hours by boat and motorbike, difficult road condition. It is also difficult to access in the rainy season because they use long off-road to reach a health facility.

***Village Health Volunteers (VHV)’s service and health education (Community)***

Health education is the solution to reducing HD. VHVs’ health education and previous project contributed to spread knowledge of the community level. and promote FD. For instance, the shift of delivery place happened around 2000, when a project was conducted and HCPs provided health education to recommend FD in the village. VHVs believe their activities contribute to making people understand FD. Some of them conducted health education at village meetings to improve the knowledge of villagers. Besides, they have no idea how they approach HD womwen who received their FD recommendation and agreed it.

***Ethnicity, religion and culture***

VHVs in one village mention that they believe *‘Pii’* (spirit) in a forest affects HD. The belief positively makes people choose FD to prevent bad thing. In other village, VHVs state that they do not conduct ritual custom for HD in the current because they go to a health facility if they feel difficult delivery.

*“People killed a pig to worship if they give a childbirth at home. But now, they don’t kill them anymore. If they feel difficult delivery, it is ‘Sabaii’ to give childbirth in the HC.”*

The culture to preparae and take plants as traditional medicine after childbirth remains in several villages.

### Societal level:

***Free delivery policy and implementation***

Women who have fear of FD cost tend to do HD even though VHVs explain it.

## Supplementary C: Questionnaire/interview guides used for the research

### Supplementary C-1: Questionnaire/interview guide for women who gave birth within one year

1. Demographic characteristics

| Participant ID: | | | Age: | | | Village: |  |
| --- | --- | --- | --- | --- | --- | --- | --- |
| Ethnic group: | | | | Religion: | | |  |
| Language: | | | | Occupation: | | |  |
| Education: | | Marriage status: | | | | Age of marriage: |  |
| Partner | Age: | | | | Language: | | |
|  | Occupation: | | | | Education: | | |
| Family member living together: | | | | | | |  |
| Property: Car (truck, van) / Motorcycle(scooter) / Agricultural machinery / other ( ) | | | | | | |  |

2. Obstetric information (ANC, delivery and PNC)

| Birth History (G P A　L D, C/S ) | | | | | |
| --- | --- | --- | --- | --- | --- |
|  | Age^1)^ | Term birth or not | Delivery Place | People who  helped you^2)^ | Complications/  abnormal symptoms ^3)^ |
| 1 |  |  |  |  |  |
| 2 |  |  |  |  |  |
| 3 |  |  |  |  |  |
| 4 |  |  |  |  |  |
| 5 |  |  |  |  |  |
| 6 |  |  |  |  |  |
| 7 |  |  |  |  |  |
| 8 |  |  |  |  |  |
| ^1)^ Mother’s age when she gave birth.  ^2)^ People who helped a woman directly; who accompanied her in facility-based delivery; who helped her directly means a person who cut the cord and took care of a child just after giving birth  ^3)^All symptoms they answered are included even though they are right or normal signs | | | | | |
| The latest pregnancy and delivery | | | | | |
| ANC visits: (times) | | | | | |
| Health check-up and visits by VHVs: (times) | | | | | |
| Health education/consultation about the delivery place: Yes / No  If yes, who gave you this information? Medical staff, VHV, other( ) | | | | | |
| Delivery:  Date of childbirth: Term birth: Yes / No ( )  Delivery place: Home / Facility ( ) / Other place ( )  Complications: Yes ( ) / No  Who helped your delivery? ( )  How did they help you? ( ) | | | | | |
| After delivery  ‘Yu Fai’: period – from day to days after delivery  Where / What they used -  Food taboo: No / Yes (The kinds of food they can/cannot eat )  Period - | | | | | |
| PNC  Mother and child receive checkup: Yes ( )days after birth / No  Care they receive: child health check/mother health check / Others ( ) | | | | | |

3. The reasons they choose the delivery place

・How was your delivery?

Whether are you satisfied with your childbirth? – Why do you think so?

・Why did you choose the place for childbirth?

Where did you want to give birth?

When did you discuss a delivery place?

What is a good/ideal delivery for you?

Why is home delivery/facility-based delivery safe/dangerous?

What are the abnormal signs which change your decision?

How did you get this knowledge?

Who gave you this knowledge?

How much money is it necessary for childbirth?

What kinds of money is necessary for facility-based delivery?

How did you prepare for childbirth?

Where did you give childbirth if you had …(money, car, etc.)?

What kinds of service/support you can get from a health facility/government / other international and national organizations?

・Who decided the place you delivery your last child?

Did you discuss the place with them before the delivery?

Whether did you decide to give birth there before your delivery?

How (with whom) did you discuss a delivery place before your delivery?

Can you say your opinion about the delivery place in your family?

Who accompanied you to the health facility (ANC, delivery and PNC) or VHV’s visit?

・How did people support you during delivery?　(family, relatives, friends, community members, medical staffs and VHVs)

Why were you satisfied/unsatisfied to their support?

・Where would you give birth if you are pregnant again?

Why do you want to give birth there?

### Supplementary C-2: Questionnaire/interview guide for Health care providers (FGD)

1. Basic information

Health facility:

| The name and type | Xepon District hospital / HC ( ) |
| --- | --- |
| The number of ANC | cases / month |
| The number of Delivery | cases / month |
| The number of assisting home delivery (if they do) | cases / month |
| The number of PNC | cases / month |

Staffs who are related to mother and child health in the facility

| Title | Number of staff | Note |
| --- | --- | --- |
| Doctor |  |  |
| Assistant Dr |  |  |
| Midwife |  |  |
| Nurse |  |  |
| Primary health staff |  |  |
| Other ( ) |  |  |

ANC

| Contents  *Please check a box if they check during ANC. | □Weight  □Length of fundus uterine / Waist circumference  □Blood pressure  □Oedema  □Fetal heartbeat  □Non-stress test (CTG)  □Urine dipstick test  □Height  □Ultrasound  □Blood check ( )  □Urine test ( )  □Else ( ) | |
| --- | --- | --- |
| Health education  / Consultation | What |  |
|  | When |  |
|  | Who |  |
|  | To whom |  |
|  | Where |  |
|  | How |  |

Delivery

| Free delivery policy  / incentives to women | | Out-of-pocket for delivery:  An incentive for women: | |
| --- | --- | --- | --- |
| People who assist delivery directly | |  | |
| Clamp and cut the cord | | Tool for cutting ( )  Sterilization: no / yes (method: ) | |
| Skin to Skin | | No / Yes (about mins) | |
| Breastfeeding | | The timing of first breastfeeding: | |
| EmOC  *Please check a box if they have these medicines and services. | | □Parenteral antibiotics  □Uterotonic drugs  □Parenteral anticonvulsants  □Manual removal of placenta  □Removal of retained products  □Assisted vaginal delivery  □Basic neonatal resuscitation | |
| Health education  / Consultation | What | |  |
|  | When | |  |
|  | Who | |  |
|  | To whom | |  |
|  | Where | |  |
|  | How | |  |

PNC

| The timing | Facility-based delivery: ( days after delivery)  Home delivery: ( days after delivery) |
| --- | --- |
| Contents | Mothers’ check-up ( )  Childs’ check-up ( )  Vaccination (Child) ( ) |

2. FGD

Date: Time: (Start) (End)

Meeting place:

Health providers who join FGD:

|  | age | title | Carrier(year) | Ethnic group |
| --- | --- | --- | --- | --- |
| 1 |  |  |  |  |
| 2 |  |  |  |  |
| 3 |  |  |  |  |
| 4 |  |  |  |  |
| 5 |  |  |  |  |

How do you think about home delivery and facility-based delivery?

How do women and their family decide delivery place?

What is ideal/good childbirth for you (Medical staffs)?

What is ideal/good childbirth for mothers and their family?

Who decides delivery place in their family?

How do you change to improve the current situation for ideal delivery?

What kinds of supports/service/approach do we need to improve for the ideal child?

### Supplementary C-3: Questionnaire/interview guide for Village health volunteers (VHVs)

1. Basic information

VHV1 profile: The year to become a VHV ( )

| Age: | Sex: | Village: |
| --- | --- | --- |
| Ethnic group: | Occupation: | Education: |
| Family | ( ) children  Home delivery: children, facility: children  Dead child: Yes ( ) / No | |

VHV2 profile: The year to become a VHV ( )

| Age: | Sex: | Village: |
| --- | --- | --- |
| Ethnic group: | Occupation: | Education: |
| Family | ( ) children  Home delivery: children, facility: children  Dead child: Yes ( ) / No | |

Village character:

| Population |  |
| --- | --- |
| Ethnic group |  |
| Religion |  |
| Village with road access | Yes / No |
| Access to a village | By motorbike (All season, only dry season, impossible to access)  By car (All season, only dry season, impossible to access) |
| Access to a health facility  (Time-consuming) | How long does it take time from here to the facility: ( ) mins/hours by ( ) |
| Public transportation | Yes (bus / songthaew / tractor / other ) / No |
| Public transportation  (How often) | Every day / 1 -5 times a week / one a week / 2 times a month / one a month / every 2-3 months / less than every 2-3 months / other( ) |
| Village with electricity | Yes / No  Year ( ) |
| Village with water supply | Yes / No  Year ( ) |
| Education | Literacy rate: |
| School in a village | Primary school(grade1-3) : Yes / No  Primary school(grade 1-5) : Yes / No  Secondary school: Yes / No |
| Trend | The age of women married: ( )years old  The age of women at first birth: ( )years old |

1. Their activities in the community

What is your work as a VHV?

What kinds of health education do you provide to women and their family?

How do you approach women to encourage them to promote their health？

Did you provide health education about the delivery place?

How did you say about the delivery place?

Who listened to your health education about the delivery place?

1. The reason people choose their delivery place

How do you think about home delivery and facility-based delivery?

How do women and their family decide delivery place?

What is ideal/good childbirth for you (Medical staffs)?

What is ideal/good childbirth for mothers and their family?

Who decides delivery place in their family?

What is an ideal delivery for woman and their family?

How do you change to improve the current situation for ideal delivery?

What kinds of supports/service/approach do we need to improve for the ideal child?

## Supplementary D: Summary sheets of women’s characteristic and statements

**Case summary (20030201)**

The latest delivery place: DHP

Latest delivery: Jan.2020

Detail of latest delivery

ANC visit: 8 times

VHVs’ visit: No visit

Complication during pregnancy and delivery: No complication (No symptoms she noticed)

Health education/consultation about the delivery place: Yes (From HCPs, VHVs, parents)

Postpartum custom ‘*Yu Fai*’: Duration 10 days / Place House

Food restriction ‘*Karam kin*’: Yes

Food she eats - pig(only livestock), wild pig, chicken(only livestock), buffalo, ‘ked’ fish

The food she doesn’t eat – beef, *‘phakkha’, ‘sadpa’*, ‘*padeak*’(fish source)

Duration 5 months

Demographic characteristics

| Age: 28 | | | Village: Kadpu | |
| --- | --- | --- | --- | --- |
| Ethnicity: Lao | | | Religion: Buddhism | |
| Language: Yes | | | Occupation: Farmer | |
| Education: 6 years | | Marriage status: Marriage | | Age of first marriage: 27 |
| Husband | Age: 27 | | Language: Yes | |
|  | Occupation: Farmer | | Education: 13 years (Higher / electricity) | |
| A family member living together: 5 people (parents, husband and child) | | | | |
| Property (For transportation): Motorbike | | | | |

Obstetric information

| Birth History: G(1) P(1) A(0) L(1) D(0) *Include a latest delivery | | | | |
| --- | --- | --- | --- | --- |
|  | Age of childbirth | Delivery Place | People who  helped you | Complications/abnormal symptoms during the perinatal period |
| 1 | 28 | DHP | Doctor | Nothing |

Statement

- She actively chose FD in DHP even though the closest health facility from her village was an HC. Because she believed complete equipment and HCPs’ care including emergency treatment in DHP; There was not complete equipment in HC. She was satisfied with her FD without complaint.

- She had an opinion that HD was not good because of unsafe. (She did not explain how unsafe it was.)

- She expected some reasons for HD; no person to take them to a health facility, bad road condition, poverty, difficulty to pay transportation cost and no car ownership.

- Her father accompanied ANC and participated in it with her. She went to ANC alone by motorbike when he was busy.

- Decision-makers were her parents, but she also participated in it. They all hoped FD from the beginning. (It seemed to be nonsense that we asked whether she planned HD.)

- She prepared the MCH book, the family book (It is recorded by family members and needed for free delivery). Her father prepared materials for childbirth and a bed for postnatal care.

- Her family had a motorbike, but she asked her relative to take her to DHP for childbirth. They arranged it in advance. The relative took her to DHP. Her parents, husband accompanied her to DHP for childbirth. In the DHP, HCPs assisted her delivery and they just observed her without doing anything.

- It took one day for her childbirth. She gave childbirth at 1 am and went back home at 7 or 8 am. (She stayed for 6-7 hours after delivery.)

- There was no payment for FD. She understood it like common sense. She was explained about it in DHP.

- After delivery, the husband mainly managed fire and water. (They need boiled water for drinking and taking a shower during *‘Yu-fai’*). Her parents cooked, and her mother helped to take a shower.

- She planed delivery in HC next because she expected the second childbirth was easier than the first without a clear reason.

**Case summary (20030202)**

The latest delivery place: HC

Latest delivery: Nov.2019

Detail of latest delivery

ANC visit: 5 times

VHVs’ visit: 2 times

Complication during pregnancy and delivery: No complication (No symptoms she noticed)

Health education/consultation about the delivery place: Yes (From HCPs, VHVs)

Postpartum custom ‘*Yu Fai*’: Duration 6 days / Place House

Food restriction ‘*Karam kin*’: Yes (food she eats - chicken(livestock), ‘Miiked’ fish, Chinese water spinach; Food she doesn’t eat – beef, ‘phan’, chicken in wild, and so on)

Duration 3 months

Demographic characteristics

| Age: 22 | | | Village: Kadpu | |
| --- | --- | --- | --- | --- |
| Ethnicity: Phoutai | | | Religion: Buddhism | |
| Language: Yes | | | Occupation: Farmer | |
| Education: 5 years | | Marriage status: Marriage | | Age of first marriage: 16 |
| Husband | Age: 25 | | Language: Yes | |
|  | Occupation: Farmer | | Education: 3 years | |
| A family member living together: 9people  (Parents-in-law, sibling-in-law, husband, children) | | | | |
| Property (For transportation): Motorbike | | | | |

Obstetric information

| Birth History: G(4) P(4) A(0) L(4) D(0) *Include a latest delivery | | | | |
| --- | --- | --- | --- | --- |
|  | Age of childbirth | Delivery Place | People who  helped you | Complications/abnormal symptoms during the perinatal period |
| 1 | 17 | DHP | Doctor | Nothing |
| 2 | 19 | DHP | Doctor | Nothing |
| 3 | 20 | DHP | Doctor | [Oligohydramnios](https://ejje.weblio.jp/content/Oligohydramnios) from 7 gestational months, but there was no problem to mother and child |
| 4 | 22 | HC | Doctor | Nothing |

**Statement**

- FD is safe for mother and child. She hoped to do FD in DHP like before at first. The reason for choosing HC this time was the existence of three children of her, distance and no car ownership. She had an opinion DHP and HC was different. There are complete equipment, many doctors in DHP.

- She was satisfied with FD in HC without complaint. She evaluated childbirth in HC was normal and good although the place was different from her hope.

- Her husband accompanied her to ANC and participated in it every time.

- Female VHV recommended her to do FD.

- Decision-makers were husband, herself, mother-in-law (She said father-in-law also participated in the decision-making, but these three people seemed to be main from her saying.) She talked about the delivery place with her husband (and mother-in-law) during pregnancy. After that, he told that to his parents-in-law.

- Her family had a motorbike, but she asked her relative to take her to DHP for childbirth. They arranged it in advance. (This action was the same with another woman from the same village.) The relative took them to the health facility not only this time but also previous childbirths.

- They prepared MCH book, materials and money for childbirth during pregnancy.

- The total payment was 200,000-300,000kip; 100,000kip for gasoline, the rest was for food including treat to the relative. All payment was covered by her (family). There was no payment for FD and the doctor. (It is seven km from the village to HC. Gasoline fee was more than double compared to our calculation.)

- Family had no role during FD. They just carried and manage their belongings.

- During ‘*Yu-fai*’, husband and mother-in-law mainly supported her. Especially, newborn care was helped by mother-in-law.

- She planed FD next time.

**Case summary (20030501)**

The latest delivery place: Home (Small hut / around six meters from the main house)

Latest delivery: Mar.2019

Detail of latest delivery

ANC visit: No visit

VHVs’ visit: 3 times

Complication during pregnancy and delivery: No complication (No symptoms she noticed)

Health education/consultation about the delivery place: Yes (From husband and parents-in-law)

Postpartum custom ‘*Yu Fai*’: Duration 8 days / Place small hut

Food restriction ‘*Karam kin*’: No

Demographic characteristics

| Age: 34 | | | Village: Kaengthong nok | |
| --- | --- | --- | --- | --- |
| Ethnicity: Tri | | | Religion: Animism | |
| Language: No | | | Occupation: Farmer | |
| Education: Never | | Marriage status: Remarriage | | Age of first marriage: 19 |
| Husband | Age: 38 | | Language: No | |
|  | Occupation: Farmer | | Education: 1 year | |
| A family member living together: 6 people (Parents-in-law, husband and children) | | | | |
| Property (For transportation): Nothing | | | | |

Obstetric information

| Birth History: G(5) P(5) A(0) L(5) D(0) *Include a latest delivery | | | | |
| --- | --- | --- | --- | --- |
|  | Age of childbirth | Delivery Place | People who  helped you | Complications/abnormal symptoms during the perinatal period |
| 1 | 19 | Home | Mother-in-law, husband, a friend(s) | Nothing (No ANC visit) |
| 2 | 21 | Home | Mother-in-law, husband, a friend(s) | Nothing (No ANC visit) |
| 3 | 23 | Home | Mother-in-law, husband, a friend(s) | Nothing (No ANC visit) |
| 4 | 31 | Home | Mother-in-law, husband, a friend(s) | Nothing (No ANC visit) |
| 5 | 33 | Home | Mother-in-law, husband, a friend(s) | Nothing (No ANC visit) |

**Statements**

- She and her husband were explained to her about the delivery place and recommended FD during pregnancy. During health education, they agreed to do FD against VHV. (It was opposed to her opinion that she hoped HD.) On the other hand, they did not talk about the delivery place to each other.

- Her relative who gave childbirth at DHP talked about that to her. But she did not hope to do HD.

- She felt her previous HD (five times) were all ‘*Kud sabaii’*. In contract, she had the opinion that DHP was *‘Sabaii’* (convenient). She said the reasons for HD last time were ‘*Kud sabaii’*. and laziness to go to DHP. In the latest HD, there was no problem, and she was satisfied. She clearly stated that money and car ownership were not reasons she did HD.

- She couldn’t expect FD cost and free delivery. She heard about FD in DHP from her relative who did FD.

- She did not know about dangerous signs (even though the male VHV said he explained it to her).

- Family role during pregnancy

- Her husband did heavy work instead of her like carrying water, crushing food.

- Her family (husband or mother-in-law) recommended FD.

- Husband and father-in-law constructed the small hut around 6 to 7 gestational months.

- Woman prepared for childbirth.

- Family role during and after HD

- She caught her baby, cut the umbilical cord with a razor and delivered the placenta by herself.

- Her husband prepared materials and drinking water.

- Her husband, mother-in-law and friend entered the small hut. He and her friend(s) received her, and mother-in-law and friend(s) did newborn care like wiping and hold the baby by clothes.

- Parents-in-law cooked.

- There was no support from the father-in-law after delivery because there was the restriction that father-in-law cannot enter the small hut.

**Case summary (20030502)**

The latest delivery place: DHP

Latest delivery: Feb.2020

Detail of latest delivery

ANC visit: 1 time

VHVs’ visit: No visit

Complication during pregnancy and delivery: No complication (No symptoms she noticed)

Health education/consultation about the delivery place: Yes (From HCPs, VHVs, parents)

Postpartum custom ‘*Yu Fai*’: Duration 7 days / Place House

Food restriction ‘*Karam kin*’: No

Demographic characteristics

| Age: 20 | | | Village: Kaengthong nok | |
| --- | --- | --- | --- | --- |
| Ethnicity: Vietnamese | | | Religion: Christianity | |
| Language: No | | | Occupation: Farmer | |
| Education: 7 years | | Marriage status: Marriage | | Age of first marriage: 16 |
| Husband | Age: 21 | | Language: Yes | |
|  | Occupation: Farmer | | Education: 5 years | |
| A family member living together: 5 people (mother-in-law, husband and children) | | | | |
| Property (For transportation): Motorbike | | | | |

She is from Vietnam. Her family lives in Vietnam. She seemed to speak some Lao language and local language, but there were some difficulties to communicate with the VHV. She communicated with her husband in Vietnamese.

Obstetric information

| Birth History: G(2) P(2) A(0) L(2) D(0) *Include a latest delivery | | | | |
| --- | --- | --- | --- | --- |
|  | Age of childbirth | Delivery Place | People who  helped you | Complications/abnormal symptoms during the perinatal period |
| 1 | 18 | DHP | Doctor, nurse | Nothing |
| 2 | 20 | DHP | Doctor, nurse | Nothing |

**Statements**

- She talked about the delivery place with her husband and decided FD in advance. He was a decision-maker. The family also agreed on FD. They prepared materials like diaper and blanket, documents like MCH book and family book.

- The reason for choosing FD was fear of complications and dangerous delivery. They recognized dizziness, abdominal pain and underback pain were abnormal signs.

- FD was *‘Sabaii’*. She was satisfied with FD because of the good service and doctors existing.

- HD is difficult because there is no equipment (for childbirth), no solution to abnormal signs at home. All people would hope FD but they cannot reach a health facility.

- Her husband accompanied the ANC visit and participated in it together.

- They went to DHP by their motorbike. They visited DHP when she felt pain; she felt pain for one day and they went to DHP. She gave a childbirth two hours after reaching DHP. HCPs helped her during FD. Her husband did not help (There was no role of a husband during FD).

- She took one day for childbirth. She gave a childbirth two hours after reaching DHP. Second childbirth was easy.

- They prepared money during pregnancy. There was no payment for FD including medicines. They paid for gasoline (around 10,000kip), food during FD.

- Family role during ‘*Yu-fai*’

- Her husband managed water and fire.

- Her mother-in-law took care of the newborn.

- She and her husband were satisfied with FD in DHP. FD was (convenient in this case). They evaluated the service, equipment and existence of doctors.

- She plans FD in DHP next childbirth.

**Case summary (20031001)**

The latest delivery place: HC

Latest delivery: Mar.2020

Detail of latest delivery

ANC visit: 2 times

VHVs’ visit: No visit (Her husband is a VHV.)

Complication during pregnancy and delivery: No complication (No symptoms she noticed)

Health education/consultation about the delivery place: Yes (From HCPs, VHVs, parents)

Postpartum custom ‘*Yu Fai*’: Duration 4 days / Place House

Food restriction ‘*Karam kin*’: No

Demographic characteristics

| Age: 28 | | | Village: La-or-Kao | |
| --- | --- | --- | --- | --- |
| Ethnicity: Mangkong | | | Religion: Animism | |
| Language: Yes (we needed an interpreter. ) | | | Occupation: Farmer | |
| Education: Never | | Marriage status: Marriage | | Age of first marriage: 17 |
| Husband | Age: 37 | | Language: Yes | |
|  | Occupation: Farmer | | Education: 8 years | |
| A family member living together: 9people (Young brother-in-law, husband and children) | | | | |
| Property (For transportation): Motorbike and tractor | | | | |

Obstetric information

| Birth History: G(5) P(5) A(0) L(6) D(0) *Include a latest delivery | | | | |
| --- | --- | --- | --- | --- |
|  | Age of childbirth | Delivery Place | People who  helped you | Complications/abnormal symptoms during the perinatal period |
| 1 | 19 | Home | Parents, husband | Nothing/twin |
| 2 | 21 | HC | Doctor | Nothing |
| 3 | 23 | Home | Parents, husband | Nothing |
| 4 | 25 | HC | Doctor | Nothing |
| 5 | 28 | HC | Doctor | Nothing |

**Statements**

- The latest delivery was tired (seven days after delivery). FD was good, satisfied with HCPs’ care, and convenient. There was no problem.

- HD and FD are different. (She was only one woman with both experiences before the latest childbirth). There are HCPs to help childbirth in an HC. There is only a husband during HD. But he doesn’t know his wife (her condition / It means no knowledge like HCPs). HD is difficult because she gives childbirth by herself. She was afraid of abnormal symptoms. She thought she had to go to the HC if a haemorrhage happened.

“Delivery in the HC is ‘*Sabaii’*. Home delivery is difficult. To give childbirth by myself. ”

- On the other hand, she also said both HD and FD were good. It is ‘*Kud sabaii*’ for her to give childbirth without pain.

- In a family, there was no talk about the delivery place during pregnancy. She just tells them near childbirth. She decided on HD by herself first. She had felt strong pain for two days without childbirth. Her husband decided and took her to the HC. She gave childbirth around one hour after reaching the HC. According to her husband, it seemed the duration of labour was eight hours. (They did not mention money preparation, but) They had afforded to pay money to go to the HC. *“I thought to give childbirth at home. I felt (strong) pain for two days (but I did not give childbirth).”*

- Family role before childbirth and when she felt labour pain.

- Husband prepared materials like a diaper and Lao skirt. (preparation for HD and FD were the same.); Husband prepared wood for cord-cutting and making fire.

- Family role during and after delivery

- She stayed at a small hut when she felt labour pain in the village. Her husband and old sister (living in the same village) entered the hut and supported her. (Her husband said he was lazy to make a small hut. It is unclear the small hut was the old one with other function.

- Her husband and old sister accompanied her to HC.

- Children waited at home without an adult.

- Husband mainly helped her during ‘*Yu-fai’* by managing water and fire.

- She has no desire for more child. She plans FD in HC next time because she was afraid of death. She didn’t think about death during the latest childbirth. (It is unclear why she feels fear of dying suddenly.)

**Case summary (20031002)**

The latest delivery place: Home (Small hut)

Latest delivery: Jan.2020

Detail of latest delivery

ANC visit: 2 times

VHVs’ visit: 5 times

Complication during pregnancy and delivery: No complication (No symptoms she noticed)

Health education/consultation about the delivery place: Yes (From VHVs and husband)

Postpartum custom ‘*Yu Fai*’: Duration 3 days / Place Small hut

Food restriction ‘*Karam kin*’: No

Demographic characteristics

| Age: 38 (She is not sure her age.) | | | Village: La-or-Kao | |
| --- | --- | --- | --- | --- |
| Ethnicity: Mangkong | | | Religion: Animism | |
| Language: Yes | | | Occupation: Farmer | |
| Education: Never | | Marriage status: Remarriage | | Age of first marriage: 15 |
| Husband | Age: 26 | | Language: Yes | |
|  | Occupation: Farmer | | Education: 3 years | |
| A family member living together: 10 people  (Parents-in-law, young sibling-in-law, husband and children) | | | | |
| Property (For transportation): Motorbike and tractor | | | | |

Obstetric information

| Birth History: G(8) P(6) A(2) L(4) D(2) *Include a latest delivery | | | | |
| --- | --- | --- | --- | --- |
|  | Age of childbirth | Delivery Place | People who  helped you | Complications/abnormal symptoms during the perinatal period |
| 1 | 15 | Home | Husband | Nothing / Dead 13months |
| 2 | 18 | Home | Husband | Nothing /Dead 3 months |
| 3 | 30 | Home | Husband | Nothing |
| 4 | 31 | Home | Husband | Nothing |
| 5 | 34 | Home | Husband | Nothing |
| 6 | 38 | Home | Husband | Nothing |

She is only one woman with spontaneous abortions in this research. She experienced twice child deaths. The reason for these deaths was not sure, but she seemed not to talk about them.

**Statements**

- She was satisfied with HD because of no complication. She had no strong pain and dizziness, it was not difficult, so she just stayed at home. She would go to an HC if she had abnormal signs (tiredness, strong pain or dizziness). She did not feel fear during HD including cord-cutting.

- She had the only experience of HD. She said HD and FD were different without explanation. HD was *‘Sabaii’* because of no problem, no strong pain and easy to childbirth. She did not hope FD because HD was *‘Kud sabaii’* for her.

- She talked about the delivery place with her husband. After that, her husband decided on a small hut as a delivery place. He constructed the small hut around six gestational months. He permitted to do HD. Parents-in-law didn’t say anything about their decision.

“She told her husband when it was nearly childbirth, her husband found a place to childbirth.”

- She did not know FD cost including free delivery because of no experience with FD even though she received two times ANC and five times VHVs’ visit.

- Family role during pregnancy

- Family members (husband and parents-in-law) helped with fieldwork.

- Husband constructed a small hut as a delivery place around six gestational months.

- Family role during HD

- Family helped her with fire and water management.

- She cut the umbilical cord with wood by herself.

- The child (2months) received vaccination in the village when HC staff came.

**Case summary (20031201)**

The latest delivery place: HC

Latest delivery: July. 2019

Detail of latest delivery

ANC visit: 5 times

VHVs’ visit: 3-4 times

Complication during pregnancy and delivery: No complication (No symptoms she noticed)

Health education/consultation about the delivery place: Yes(From HCPs, VHVs, parents-in-law, husband)

Postpartum custom ‘*Yu Fai*’: Duration 12 days / Place House (kitchen)

Food restriction ‘*Karam kin*’: No

Demographic characteristics

| Age: 17 | | | Village: Kaengluang Nok | |
| --- | --- | --- | --- | --- |
| Ethnicity: Mangkong | | | Religion: Animism | |
| Language: Yes | | | Occupation: Farmer | |
| Education: 6 years | | Marriage status: Marriage | | Age of first marriage: 16 |
| Husband | Age: 19 | | Language: Yes | |
|  | Occupation: Farmer | | Education: 8 years | |
| A family member living together: 7 people  (Parents-in-law, three siblings-in-law, husband and child) | | | | |
| Property (For transportation): Motorbike | | | | |

Obstetric information

| Birth History: G(1) P(1) A(0) L(1) D(0) *Include a latest delivery | | | | |
| --- | --- | --- | --- | --- |
|  | Age of childbirth | Delivery Place | People who  helped you | Complications/abnormal symptoms during the perinatal period |
| 1 | 16 | HC | HCPs | Nothing |

**Statements**

- She decided her own partner and marriage by herself. Teenage marriage is common.

- Her husband and herself decided on FD in the HC during ANC.

*“I say when I feel labour pain and then go to HC for a childbirth”*

- Her family prepared money (1,000,000kip) and a family book for FD. She did not prepare materials like clothes and diapers for childbirth. She bought them in (or around) HC. Her husband managed money and she did not know the details. He prepared money by selling livestock. She guessed he paid money for waiting for childbirth, water, child goods like a diaper. She was not sure whether her husband pays money for medicines because she had no idea of FD cost and free delivery.

- She was satisfied with FD. It was good because of the HCPs’ care. She had the opinion that HD and FD were different; FD was good because there were medical staffs and doctor to help in HC. People would do HD if it were easy delivery. People would do FD if it was difficult. She was afraid of a difficult delivery.

- She felt labour pain in the morning. Her husband took her to the HC with her old sister by motorbike. They reached the HC at 7 am. She gave childbirth at 1 pm. It took around 6 hours for childbirth.

- Family role during and after delivery

- Husband and old sister accompanied to HC.

- They had no role for FD, just received her and encourage her.

- Husband mainly helped her including fire management and washing.

- Mother-in-law took care of the newborn.

- Father-in-law didn’t help because of restriction.

- Relatives helped her directly like water preparation for a shower. Commonly, women helped her except for a husband.

- Friends and neighbour advised her and their family. (They didn’t enter the small hut.)

- She followed health education from HCP after delivery; not to take a shower for three days; not to give a meal to a child. According to the research assistant, shower restriction was far from her sense. There was no health education about PNC. (We were not sure she misunderstood health education or an HCP taught that exactly.) Relatives also didn’t say anything about PNC.

- Her child (eight months) is healthy. She gave her mother’s milk and meal.

**Case summary (20031202)**

The latest delivery place: Home (main house, not small hut)

Latest delivery: Jan. 2020

Detail of latest delivery

ANC visit: 4-5 times

VHVs’ visit: 4-5 times

Complication during pregnancy and delivery: No complication (No symptoms she noticed)

Health education/consultation about the delivery place: Yes (From HCPs, VHVs, relatives)

Postpartum custom ‘*Yu Fai*’: Duration 10 days / Place House (kitchen)

Food restriction ‘*Karam kin*’: No

Demographic characteristics

| Age: 36 | | | Village: Kaengluang Nok | |
| --- | --- | --- | --- | --- |
| Ethnicity: Mangkong | | | Religion: Animism | |
| Language: No | | | Occupation: Farmer | |
| Education: Never | | Marriage status: Marriage | | Age of first marriage: 18 |
| Husband | Age: 40 | | Language: No | |
|  | Occupation: Farmer | | Education: Never | |
| A family member living together: 8 people (husband and children) | | | | |
| Property (For transportation): Motorbike | | | | |

Obstetric information

| Birth History: G(8) P(8) A(0) L(6) D(2) *Include a latest delivery | | | | |
| --- | --- | --- | --- | --- |
|  | Age of childbirth | Delivery Place | People who  helped you | Complications/abnormal symptoms during the perinatal period |
| 1 | 19 | Home | Husband | Nothing |
| 2 | 21 | Home | Husband, relative | Nothing / dead two months |
| 3 | 22 | Home | Husband, relative | Nothing |
| 4 | 23 | Home | Husband, relative | Nothing/ dead two months |
| 5 | 24 | Home | Husband, relative | Nothing |
| 6 | 31 | Home | Husband, relative | Nothing |
| 7 | 33 | Home | Husband, relative | Nothing |
| 8 | 36 | Home | Husband, relative | Nothing |

She was not sure of the reasons for child deaths.

**Statements**

- Reasons for HD were no money, no car. It was impossible to go to a health facility for childbirth by motorbike because of road condition (9E road / 35mins by motorbike). Easiness of childbirth was also another reason for HD. She expected many people did HD because they cannot do FD.

- She had no idea of dealing with an abnormal symptom like a haemorrhage. She thought she should go to a health facility if she had abnormal symptoms like remained placenta and/or haemorrhage. (There is the possibility she says haemorrhage because of conversation between VHV and interviewer. ) Some friends did FD. She thought FD was good.

- Husband asked her whether she went to DHP for childbirth. She rejected the suggestion because of *‘Kud sabaii’*. She experienced HD without complication. These were all *‘Kud sabaii’*. She decided on HD by herself. Relatives said no need to be afraid of HD because of *‘Kud sabaii’*

- The latest HD was good because of no fever. There was no difficulty. The duration of labour was four to five hours. It was easy childbirth for her because she just felt pain, no tired and no complication.

- Family role

- Husband or child accompanied her to ANC.

- Husband received her during delivery.

- Relatives entered a room and encouraged her.

- Woman cut the umbilical cord with bamboo.

- Daughter managed water and cooked during and after delivery.

- Husband and daughter prepared clothes.

- She estimated the cost for FD was 400,000 or 500,000 kip. It included gasoline fee, material consumption and *‘Khamu’* (unofficial payment to HCPs). The expected cost was higher than the cost we calculated together (around 100,000 kip except for food and *‘Khamu’* ). (According to female VHV, she was afraid of payment.)

- Her daughter did HD twice. She helped her daughter’s HD.

- She hopes for FD next even though she has no desire for more child.

**Case summary (20031203)**

The latest delivery place: HC

Latest delivery: Dec.2019

Detail of latest delivery

ANC visit: No visit

VHVs’ visit: No visit

Complication during pregnancy and delivery: No complication (No symptoms she noticed)

Health education/consultation about the delivery place: Yes (From HCPs, VHVs)

Postpartum custom ‘*Yu Fai*’: Duration 10 days / Place House (kitchen)

Food restriction ‘*Karam kin*’: No

Demographic characteristics

| Age: 20 | | | Village: Alai Noy | |
| --- | --- | --- | --- | --- |
| Ethnicity: Tri | | | Religion: Animism | |
| Language: No | | | Occupation: Farmer | |
| Education: Never | | Marriage status: Marriage | | Age of first marriage: 18 |
| Husband | Age: 20 | | Language: No | |
|  | Occupation: Farmer | | Education: Never | |
| A family member living together: 9 people  (Parents-in-law, young siblings-in-law, husband and child) | | | | |
| Property (For transportation): Motorbike | | | | |

Obstetric information

| Birth History: G(1) P(1) A(0) L(1) D(0) *Include a latest delivery | | | | |
| --- | --- | --- | --- | --- |
|  | Age of childbirth | Delivery Place | People who  helped you | Complications/abnormal symptoms during the perinatal period |
| 1 | 20 | HC | HCPs | Nothing |

**Statements**

- Mother-in-law said to do FD because of fear of difficult childbirth. She didn’t feel anything to her opinion and just followed it.

- She had the same opinion as other villagers that women did not need to go to ANC if there was no symptom. She said busyness was a reason not to visit ANC. The mother-in-law (decision-maker) did not say anything about the absence of ANC visit. She just confirmed a woman’s condition without a symptom like a fever.

- She was satisfied with FD. It was painful, but she felt just happy when the baby came out. There was no complaint to FD. FD is more *‘Sabaii’* and easy because there are HCPs to help if a complication happens in HC.

- They paid a gasoline fee (100,000kip for a round-trip) to a car owner to take her to HC. The car owner was not her relative. In this village, people ask a car owner’s favour to take them to a health facility by paying a gasoline fee in an emergency. The family prepared money by day labour of cassava harvesting.

- Cost for FD was; gasoline fee (no payment for car rent) 100,000kip, unofficial payment to HCP 80,000kip. There is the possibility to pay it as unofficial payment and material set, 45,000kip. The research assistant and VHV finish this theme before asking for the details.

- They went to the HC around 7 pm as she felt labour pain. She gave childbirth at 7 am (around 12 hours after leaving for HC). She went back home one hour after delivery.

- Family and relatives’ role during and after delivery

- Husband, mother-in-law and relatives (older persons) who had the experience of childbirth(s), ten people in total companied her to HC for childbirth by car.

- Family received her during delivery. Nurse(s) provided care.

- Husband and mother-in-law managed water and fire including collecting woods after delivery.

- Husband cooked.

- She took a shower by herself.

- Father-in-law didn’t do anything (restriction).

- Mother-in-law took care of the child, especially after she restarted fieldwork (three months after delivery)

- She hopes for FD next pregnancy.

**Case summary (20031204)**

The latest delivery place: Home

Latest delivery: Nov.2019

Detail of latest delivery

ANC visit: No visit

VHVs’ visit: 4-5 times

Complication during pregnancy and delivery: No complication (No symptoms she noticed)

Health education/consultation about the delivery place: Yes (From HCPs, VHVs, relatives)

Postpartum custom ‘*Yu Fai*’: Duration 3 days / Place House (kitchen)

Food restriction ‘*Karam kin*’: No

Demographic characteristics

| Age: 37 | | | Village: Alai Noy | |
| --- | --- | --- | --- | --- |
| Ethnicity: Tri | | | Religion: Animism | |
| Language: No | | | Occupation: Day laborer | |
| Education: Never | | Marriage status: Marriage | | Age of the first marriage:17 |
| Husband | Age: 40 | | Language: No | |
|  | Occupation: Day laborer | | Education: Never | |
| A family member living together: 6 people (husband and children) | | | | |
| Property (For transportation): Nothing | | | | |

She understood half of the interview in the Lao language.

Her family moved from the mountain five years ago. There is no kin in the current village.

Obstetric information

| Birth History: G(6) P(6) A(0) L(6) D(0) *Include a latest delivery | | | | |
| --- | --- | --- | --- | --- |
|  | Age of childbirth | Delivery Place | People who  helped you | Complications/abnormal symptoms during the perinatal period |
| 1 | 20 | Home | Relative(s) | Nothing |
| 2 | 24 | Home | Relative(s) | Nothing |
| 3 | 26 | Home | Relative(s) | Nothing |
| 4 | 28 | Home | Relative(s) | Nothing |
| 5 | 31 | Home | Relative(s) | Nothing |
| 6 | 37 | Home | Friends | Nothing |

**Statements**

- There is no filed for the family. They rely on cash to buy food and everything. Both couples are day labourers (50,000kip/person/day). The first son also works as a day labourer sometimes. Working time is from 7 am to 5 pm including 2 hours lunch break. She continued the labour during pregnancy and restarted the work on the 4^th^ days after delivery.

- She did not receive ANC because of laziness to go. She knew free delivery (not to pay for FD).

- She did not talk about the delivery place with family. She thought FD was better, but she did HD because of *‘Kud Sabaii’,* no difficulty, not painful and no complication. HD was easier than FD for her. The duration of labour was less than one hour. She had the opinion that she would go to DHP if it was a difficult delivery. Abnormal symptoms she knew were convulsion, strong pain and fever.

“I didn’t go to the HP. It wasn’t painful, with no fever. Only I gave birth. (The baby) came out.”

- Family and others’ role during delivery

- Four or five female friends entered the delivery room and helped her by receiving. They went back home after childbirth.

- Husband was lazy. She said the only husband managed fire during delivery.

- Woman received a baby, cut the umbilical cord with bamboo, delivered the placenta, cleaned, wiped and held a baby by herself.

-

- Family role after delivery.

- Children managed water and fire.

- Husband was lazy. He cooked and managed fire also. He helped normally.

- Husband and children take care of a newborn when she goes to work. They give artificial milk.

- Child received vaccination at the village three months after birth.

- Her perception of contraceptive methods is uncomfortable because of the side effect. She has experience with injection.

- She plans HD if it is easy next childbirth.

**Case summary (20031601)**

The latest delivery: Home (the corner of the kitchen) with husband

Latest delivery: Oct.2019

Detail of latest delivery

ANC visit: No ANC visit

VHVs’ visit: 3-4 times

Complication during pregnancy and delivery: No complication (No symptoms she noticed)

Health education/consultation about the delivery place: Yes (From HCPs, VHVs, relatives)

Postpartum custom ‘*Yu Fai*’: Duration 3days / Place House (kitchen)

Food restriction ‘*Karam kin*’: No

Demographic characteristics

| Age: 33 | | | Village: Kalouk mai-kao | |
| --- | --- | --- | --- | --- |
| Ethnicity: Tri | | | Religion: Animism | |
| Language: No | | | Occupation: Farmer | |
| Education: Never | | Marriage status: Marriage | | Age of first marriage: 23 |
| Husband | Age: 23 | | Language: Yes | |
|  | Occupation: Farmer | | Education: 4 years | |
| A family member living together: 6people (husband and children) | | | | |
| Property (For transportation): Noting | | | | |

Birth History

| G(5) P(5) A(0) L(4) D(1) *Include a latest delivery | | | | |
| --- | --- | --- | --- | --- |
|  | Age of childbirth | Delivery Place | People who  helped you | Complications/abnormal symptoms during the perinatal period |
| 1 | 24 | Home | Husband | Normal / Dead 2 days after delivery |
| 2 | 25 | Home | Husband | Normal |
| 3 | 28 | Home | Husband | Normal |
| 4 | 30 | Home | Husband | Normal |
| 5 | 33 | Home | Husband | Normal |

She was not sure about the cause of her first child death (2days after delivery). She expressed she had no complication; the childbirth itself was normal even though its an experience.

**Statements**

- HD was normal without any abnormal symptom latest time.

- She and her husband said to wanted to go to DHP because FD is ‘*Sabaii*’ because they trusted in treatment of complication in DHP including prolonged delivery *“If the baby is not born, the doctor has injection medicine”.* They thought HD and FD were different. There is no medicine, HCP and medicine at home. HD includes the risk of maternal and child died because there is no way if the problem happens at home. But there was no money, no ownership of a motorbike and a short time for childbirth (less than one hour), so they did not reach DHP (the closest health facility from her village).

- On the other hand, she said the opposite opinion to FD. For instance, “It is ok to stay at home when a child is healthy.” “I wouldn’t go (to the health facility when it took 2-3 days for childbirth).”

- All her childbirths were ‘*Kud sabaii*’ and no problem for her even though neonatal death happened in the past. The husband also said there were good delivery and nothing happened.

- They expected cost for FD was around 200,000-300,000kip for meals, water and gasoline. There was no money preparation because of busyness(fieldwork).

- No preparation for childbirth before delivery like washing clothes and diapers.

- She felt labour pain around 8 am. Her husband stayed with her without looking for transportation mean like asking for a relative’s motorbike. There seemed to be no relatives at the time because of fieldwork by motorbike from 7-8 am. During HD, the husband received her, caught the baby, cut the umbilical cord with a new razor and water/fire management. She wiped the baby’s body. Husband washed the baby with soap immediate after delivery.

- There was no HCPs’ visit after delivery.

- She hopes to give next childbirth at home if she is pregnant again (no desire of more child).

- Her knowledge of abnormal symptom was only fetus moving. She received health education from VHV and participated in a village meeting, but she didn’t remember other symptoms.

**Case summary (20031602)**

The latest delivery place: DHP

Latest delivery: Jan. 2020

Detail of latest delivery

ANC visit: 4 times VHVs’ visit: 4-5 times

Complication during pregnancy and delivery: No complication (No symptoms she noticed)

Health education/consultation about the delivery place: Yes (From HCPs, VHVs, relatives)

Postpartum custom ‘*Yu Fai*’: Duration 3days / Place House(kitchen)

Food restriction ‘*Karam kin*’: No

Demographic characteristics

| Age: 36 | | | Village: Kalouk mai-kao | |
| --- | --- | --- | --- | --- |
| Ethnicity: Tri | | | Religion: Christianity | |
| Language: No  (she answered sometimes in the Lao language) | | | Occupation: Farmer | |
| Education: Never | | Marriage status: Marriage | | Age of first marriage: 15 |
| Husband | Age: 36 | | Language: No | |
|  | Occupation: Farmer | | Education: Never | |
| A family member living together: 7 people (husband and children) | | | | |
| Property (For transportation): Noting | | | | |

Obstetric information

| Birth History: G(9) P(9) A(0) L(5) D(4) *Include a latest delivery | | | | |
| --- | --- | --- | --- | --- |
|  | Age of childbirth | Delivery Place | People who  helped you | Complications/abnormal symptoms during the perinatal period |
| 1 | 15 | Home | Husband | Normal / Dead (no detail) |
| 2 | 16 | Home | Husband | Normal / Dead (no detail) |
| 3 | 18 | Home | Husband | Normal / Dead (no detail) |
| 4 | 19 | Home | Husband | Normal / Dead (no detail) |
| 5 | 21 | Home | Husband | Normal |
| 6 | 22 | Home | Husband | Normal |
| 7 | 23 | Home | Husband | Normal |
| 8 | 33 | Home | Husband | Normal |
| 9 | 36 | DHP | Doctor | Normal |

She lost four children (1^st^ to 4^th^ children). She did not know these causes and the age they die. Just she said she raised them, so the interviewer explained to the author it may not neonatal death.

**Her statements**

- She did not talk about the delivery place with her husband during pregnancy. She told him when she felt labour pain. She did not plan FD because of no money. She was afraid of childbirth on the road. The previous childbirths happened suddenly and easily, so she could not reach an HC. These were *‘Sabaii’*’ because of no complication. Therefore, she preferred HD. She recognized difficulty to use a motorbike during labour pain. She did not know abnormal symptoms.

- In the latest childbirth, there was no childbirth although she felt painful for 2-3days. She decided to do HC by herself. Her old sister and her husband (old brother-in-law) lived near her village and owned a car. They called her and took her to DHP. (It was unclear which was first, she asked or old sister’s couple proposed.) They gave transportation and financial support to her. She was not sure how the couple earned. It may be selling something.

- Total cost for childbirth was 500,000kip (gasoline 300,000kip, food and water to all companions)

It is unclear how much the old site couple paid, but the majority of the cost would be covered by the couple. She and her husband did not have enough money.

- Many people accompanied her to DHP; husband, old sister, young sibling(s), children. On the other hand, she explained her children did not accompany her to DHP. She stayed at DHP for one day to give childbirth.

- Family role during and after delivery’

- Husband stayed with her without going to the field after she felt labour pain.

- Husband and children managed water and fire. And the children cooked.

- Her daughter (first child) took care of the baby during the daytime (from 6 am to 3 pm).

- She restarted fieldwork with her husband after finishing Yu-fai care(3 days after delivery). They gave the mother’s milk and porridge to the baby.

- Most children stay at home (one son has been studying) and take care of the baby and each other when the parents go to the filed. (age of children are around 15, 14, 13, 3years old and the baby)

- She wants two more children. She hopes FD in DHP next pregnancy.

**Case summary (20031801)**

The latest delivery place: HC

Latest delivery: Feb.2020

Detail of latest delivery

ANC visit: 4 times

VHVs’ visit: 2 times

Complication during pregnancy and delivery: No complication (No symptoms she noticed)

Health education/consultation about the delivery place: Yes (From HCPs, VHVs, parents)

Postpartum custom ‘*Yu Fai*’: Duration 5days / Place House (kitchen)

Food restriction ‘*Karam kin*’: No

Demographic characteristics

| Age: 23 | | | Village: Vangmorthoum | |
| --- | --- | --- | --- | --- |
| Ethnicity: Tri | | | Religion: Animism | |
| Language: Yes | | | Occupation: Farmer | |
| Education: 8 years | | Marriage status: Marriage | | Age of first marriage: 18 |
| Husband | Age: 25 | | Language: Yes | |
|  | Occupation: Day laborer | | Education: 7 years | |
| A family member living together: 7 people (parents-in-law, husband and children) | | | | |
| Property (For transportation): Motorbike and tractor | | | | |

Obstetric information

| Birth History: G(3) P(3) A(0) L(3) D(0) *Include a latest delivery | | | | |
| --- | --- | --- | --- | --- |
|  | Age of childbirth | Delivery Place | People who  helped you | Complications/abnormal symptoms during the perinatal period |
| 1 | 18 | HC | Doctor | Nothing |
| 2 | 21 | HC | Doctor | Nothing |
| 3 | 23 | HC | Doctor | Nothing |

**Her statement**

- FD (in HC) was good, *‘Sabaii’* and not difficult. FD and HD are different. In an HC, she can receive anything including injection (she did not remember what medicines she received), and there are many things like plastic bags. She thinks HD is difficult. There are not many things like medicines at home.

- She talked about the delivery place with her husband around 6months gestational age. Husband asked her preference of delivery place (DHP, HC or home), so she chose FD by herself. Her husband agreed to it. Decision-makers were herself and her husband. Parents-in-law did not participate in the decision-making. They told her that FD in HC was *Sabaii’.* She and all family members hoped for FD from the beginning.

- Her husband took her to the HC by his tractor. It took 10-15mins. The road (from her village to the HC) is good. Her mother-in-law and young sister-in-law (a wife of her young brother) accompanied her.

- In the HC, only HCPs assisted her. Mother-in-law and young sister-in-law waited outside of the delivery room. Her husband entered the room and just stood behind her to encourage her.

- She went back home one or two hours after delivery. It was after finishing the transfusion. According to an HC staff, the family started preparing their tractor as she gave childbirth. (It is common to stay shortly after delivery in the closest HC).

- Family role during ‘Yu-fai’

- Her husband managed water and fire.

- Parents-in-law did not help directly. They cooked.

- Mother-in-law took care of the baby (shower).

- Everyone can enter the postnatal room without restriction in this family.

- It was three weeks after delivery. She stays at home and concentrates on the care of three children without going to the filed.

- She gave the baby mother’s milk and porridge 3 times a day. She thought mother’s milk was not enough especially when she would restart fieldwork. (Against the suggestion not to give it until 6 months) “Oh, 6 months I would go work to the filed. Who goes to work?”

- The number of children (three daughters) is enough for her. But she hopes for one male child.

**Case summary (20031802)**

The latest delivery place: Home (the corner of a small hut)

Latest delivery: Jan.2020

Detail of latest delivery

ANC visit: 2 times

VHVs’ visit: 2 times

Complication during pregnancy and delivery: Anemia

Health education/consultation about the delivery place: Yes (From HCPs, VHVs, parents)

Postpartum custom ‘*Yu Fai*’: Duration 6 days /

Place (first 3days) small hut (next 3days) kitchen

Food restriction ‘*Karam kin*’: No

Demographic characteristics

| Age: 25 | | | Village: Vangmorthoum | |
| --- | --- | --- | --- | --- |
| Ethnicity: Tri | | | Religion: Animism | |
| Language: Yes | | | Occupation: Farmer | |
| Education: Never | | Marriage status: Remarriage | | Age of first marriage: 15 |
| Husband | Age: 35 | | Language: Yes | |
|  | Occupation: Farmer | | Education: Never | |
| A family member living together: 7people (mother-in-law, husband and children) | | | | |
| Property (For transportation): Motorbike and tractor | | | | |

*She moved from the village in the mountain to this village when she remarried 2 years ago. It was my first childbirth after moving.

* She understood less the Lao language compared to another literate woman.

Obstetric information

| Birth History: G(3) P(3) A(0) L(2) D(1) *Include a latest delivery | | | | |
| --- | --- | --- | --- | --- |
|  | Age of childbirth | Delivery Place | People who  helped you | Complications/abnormal symptoms during the perinatal period |
| 1 | 17 | Home | Husband | Nothing / the child was dead 2 months after delivery (fever and throat sore) |
| 2 | 22 | Home | Uncle | Nothing |
| 3 | 25 | Home | Husband | Nothing |

**Her statement**

- Around four months of gestational age, she felt abdominal pain. Her husband decided and took her to the DHP and she was diagnosed with suspected appendicitis. However, she was diagnosed with only anaemia finally in Savannakhet provincial hospital. After that, she had no symptoms.

- She talked about the delivery place with her husband in advance. She asked her husband where she would give childbirth. He proposed to take her to the HC, but she hoped HD because of *‘Sabaii’* and shyness. Her husband accepted her decision.

*“He said he would take me to the HC for childbirth. I said I didn’t want to go.”*

- Her husband supported HD. He constructed a small hut for HD behind the main house. It took 3 days.

*“She asked her husband where she would give childbirth. He said he would take her to the HC for childbirth. She said she didn’t want to go.”*

- First, she agreed that she had to go to the health facility if there was an abnormal symptom. But she changed her statement that she wouldn’t want to go to the HC if complication like postpartum haemorrhage happened because of shyness. She expected women with FD were not shy.

- HD was good. She did HD because it was *‘Sabaii’*, so she did not reach a health facility. The duration of labour was around one hour. She felt labour pain at the main house to sleep. She walked to the small hut and gave childbirth by squatting at the corner of the small hut at 4 am.

- Family support during delivery

- Husband assisted HD. He received her, cut the umbilical cord with wood.

- She received the baby, wipe its body and clean blood by herself.

- Mother-in-law makes the newborn take a shower just after delivery.

- She is not sure whether she desires more child. Her husband has no hope. She hopes for HD for her next pregnancy because of the easiness of childbirth.

**Case summary (20031803)**

The latest delivery place: Home (small hut)

Latest delivery: Mar.2020

Detail of latest delivery

ANC visit: 1 time

VHVs’ visit: No visit

Complication during pregnancy and delivery: No complication (No symptoms she noticed)

Health education/consultation about the delivery place: No

Postpartum custom ‘*Yu Fai*’: Duration 9 days (Plan; It was 6days after delivery on that day)

Place House(kitchen)

Food restriction ‘*Karam kin*’: Yes (food she eats - vegetables, chicken, fish, bamboo shoots

The food she doesn’t eat - pork, frog, shrimp, shellfish, eel)

Duration Not sure (she is following the advice of mother-in-law)

Demographic characteristics

| Age: 17 | | | Village: Poung | |
| --- | --- | --- | --- | --- |
| Ethnicity: Tri | | | Religion: Buddhism | |
| Language: Yes | | | Occupation: Farmer | |
| Education: 6 years | | Marriage status: Marriage | | Age of first marriage: 16 |
| Husband | Age: 19 | | Language: Yes | |
|  | Occupation: Day laborer | | Education: 7 years | |
| Family member living together:7people  (mother-in-law, siblings-in-law, husband and child) | | | | |
| Property (For transportation): Motorbike | | | | |

Obstetric information

| Birth History: G(1) P(1) A(0) L(1) D(0) *Include a latest delivery | | | | |
| --- | --- | --- | --- | --- |
|  | Age of childbirth | Delivery Place | People who  helped you | Complications/abnormal symptoms during the perinatal period |
| 1 | 17 | Home | Mother-in-law, husband | Nothing |

She didn’t receive PNC, but her baby received vaccination by an HC staff (neighbour) at home.

**Her statements**

- She visited ANC once around six months without symptom. Her husband decided ANC visit, and her young sister-in-law accompanied her. She did not go second ANC visit because there was no vehicle during day time (her husband went to work by motorbike). She said she did not receive health education about childbirth and delivery place. She had never heard about mother and child death.

- She did not talk about the delivery place with her family. She did not plan the delivery place. She just wanted to give childbirth to an HC in her mind because she listened FD was *‘Sabaii’*, and there was no person to help at home.

- She did not prepare for childbirth because of restriction. (According to the HC staff in this area, the majority of them do not follow this restriction, but still, some people believe and conduct it. People believe the baby would die if they prepare stuff before childbirth.)

- She said the reason for HD was no person to take her to the HC. The duration of labour was around six hours (from midnight, midnight to 6 am). She was in a small hut with her husband when she started feeling labour pain. It was not sure when it was constructed, but the couple used it for sleeping. She explained only her pain to her husband. He expected it was abdominal pain because of spicy food consumption. She hoped to be taken to the HC, but she did not ask clearly. She felt angry with his reaction. She stayed and gave childbirth at the small hut in the morning. She felt tired after delivery. She had no feeling about the gap between her hope of FD and HD implementation.

*“I tried to say that but my husband didn’t understand.”*

*“I was painful, tired to say. My husband said that I ate too much spicy food.”*

*“Only* *I said ‘I have abdominal pain, strong pain’.”*

*“I feel angry. I feel angry with my husband.”*

- Family role during delivery

- Husband and mother-in-law entered the small hut and assisted delivery.

- Husband received her.

- Mother-in-law received the baby, cut the umbilical cord and assisted placenta delivery (it came out spontaneously).

- She cleaned her body by herself.

- Her aunt prepared wood for cord-cutting.

- The baby was taken a shower immediately.

- She was conducting Yu-fai and food restriction by following the advice from the mother-in-law. She has been drinking *‘Hakmai’* now (with doing *‘Yu-fai’*)

- Family role after delivery

- Husband and mother-in-law cooked.

- Mother-in-law took care of the child.

- Siblings-in-law did not do anything.

- She plans to do HD if she is pregnant again.

*“I would do delivery at home because I gave birth first child at home. The second child should be given birth at home as well.”*

**Case summary (20031804)**

The latest delivery place: DHP

Latest delivery: Jun.2019

Detail of latest delivery

ANC visit: 5 times (in HC)

VHVs’ visit: 4 times

Complication during pregnancy and delivery: No complication (No symptoms she noticed)

Health education/consultation about the delivery place: Yes (From HCPs, VHVs, mother)

Postpartum custom ‘*Yu Fai*’: Duration 10 days / Place House(kitchen)

Food restriction ‘*Karam kin*’: Yes (food she eats - vegetables, fish, chicken, frog

The food she doesn’t eat – pickles, spicy foods)

Duration 3 months

Demographic characteristics

| Age: 22 | | | Village: Poung | |
| --- | --- | --- | --- | --- |
| Ethnicity: Lao | | | Religion: Buddhism | |
| Language: Yes | | | Occupation: Farmer | |
| Education: 5 years | | Marriage status: Divorce  (after delivery) | | Age of first marriage: 19 |
| Husband | Age: 29 | | Language: Yes | |
|  | Occupation: Day laborer | | Education: 5 years | |
| A family member living together: 4 people (mother, young brother and child) | | | | |
| Property (For transportation): Noting | | | | |

Obstetric information

| Birth History: G(2) P(2) A(0) L(1) D(1) *Include a latest delivery | | | | |
| --- | --- | --- | --- | --- |
|  | Age of childbirth | Delivery Place | People who  helped you | Complications/abnormal symptoms during the perinatal period |
| 1 | 19 | DHP | Doctor, nurse | Stillbirth 24weeks of pregnancy |
| 2 | 21 | DHP | Doctor, nurse | Nothing |

(Stillbirth) She had no ANC visit before noticing it. There was no symptom before the health facility visit.

**Her statements**

- HC and DHP are different. She evaluates HC has modern machines, HCPs to take care of ANC. On the other hand, she expresses more trust in DHP, its safety, HCP’s care.

“HCPs are different so much. The equipment is completed, many kinds (of equipment). They come to give much suggestion.”

“HCPs took good care and did monitoring well when I felt pain.”

- She actively chose FD in DHP by herself even though it was not the closest health facility to her village. She was afraid of childbirth because of stillbirth. She talked with the family especially the mother about the delivery place including transportation mean. They asked the friend of her mother to take her to DHP during pregnancy.

*“I talked where I would go for my childbirth, how we would go.”*

- The friend of her mother took them to the DHP. She did not pay.

- She did not pay for FD (free delivery).

- They went to the DHP one hours after the start of feeling labour pain. The duration of labour was 18 hours. She stayed in a patient room without going out before childbirth. Her husband, mother and a friend waited at DHP. They did not enter the delivery room.

- During *‘Yu-fai’*, husband and mother helped together including newborn care. Especially, the mother took care of the child when she went to the field (three or four months after delivery).

- She drank ‘Hakmai(ຮາກໄມ້)’ during ‘Yu-fai’.

- She hopes FD in DHP next childbirth because of the highest safe delivery and cares even though she has no willing of marriage and more child.

## Supplementary G: Ethical approval

### Supplementary G-1: Nagasaki university ethical approval


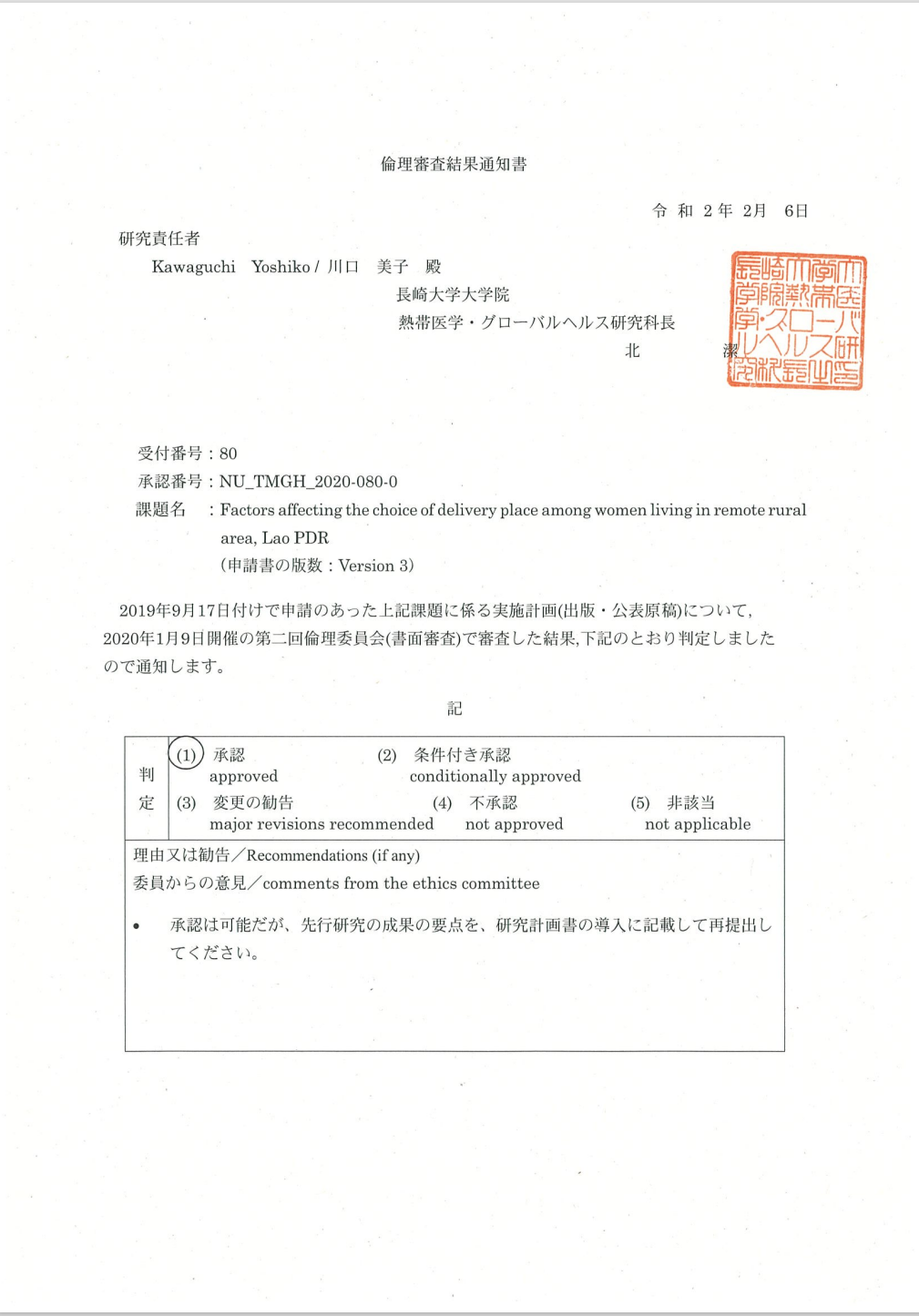


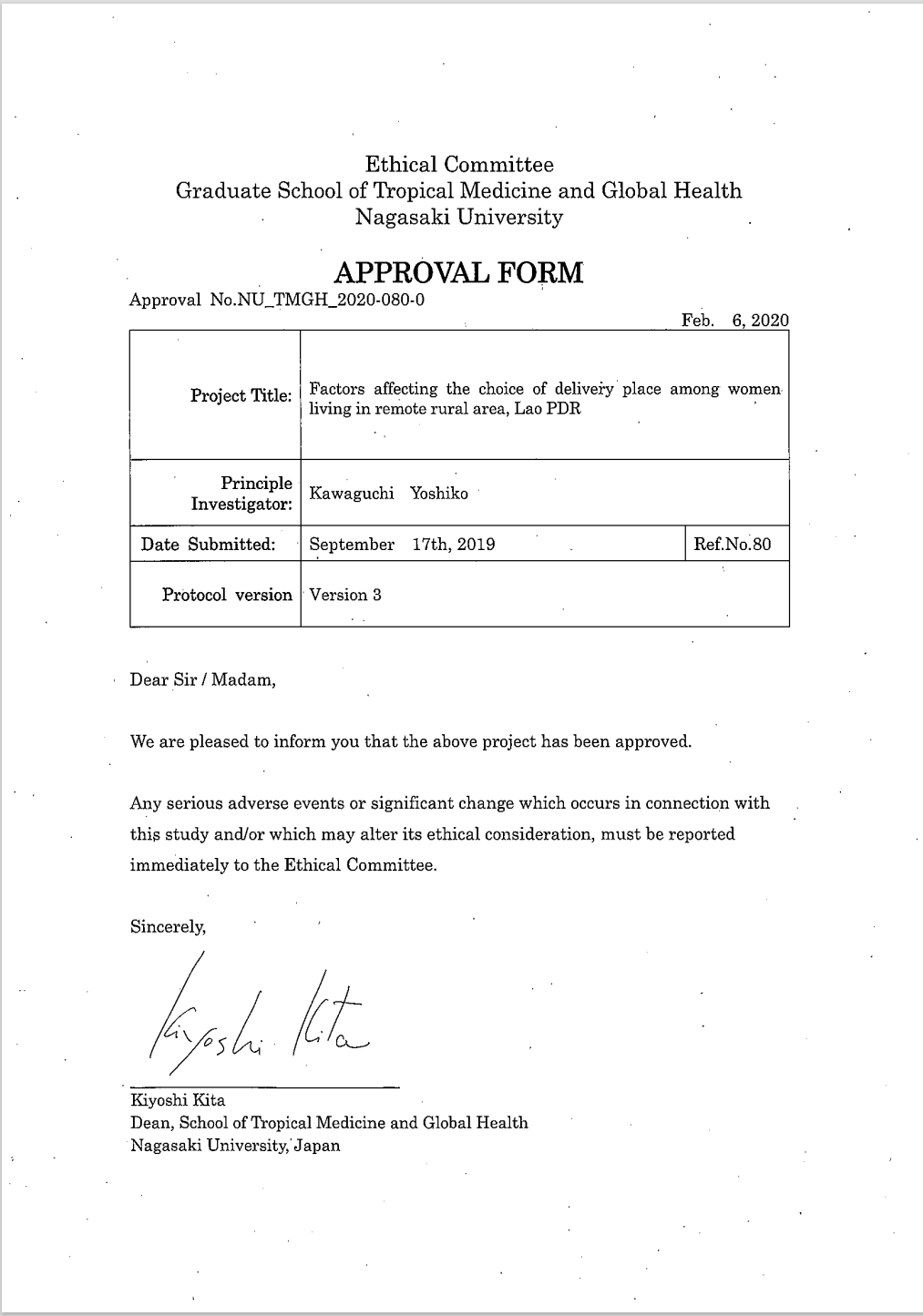


### Supplementary G-2: Local ethical approval


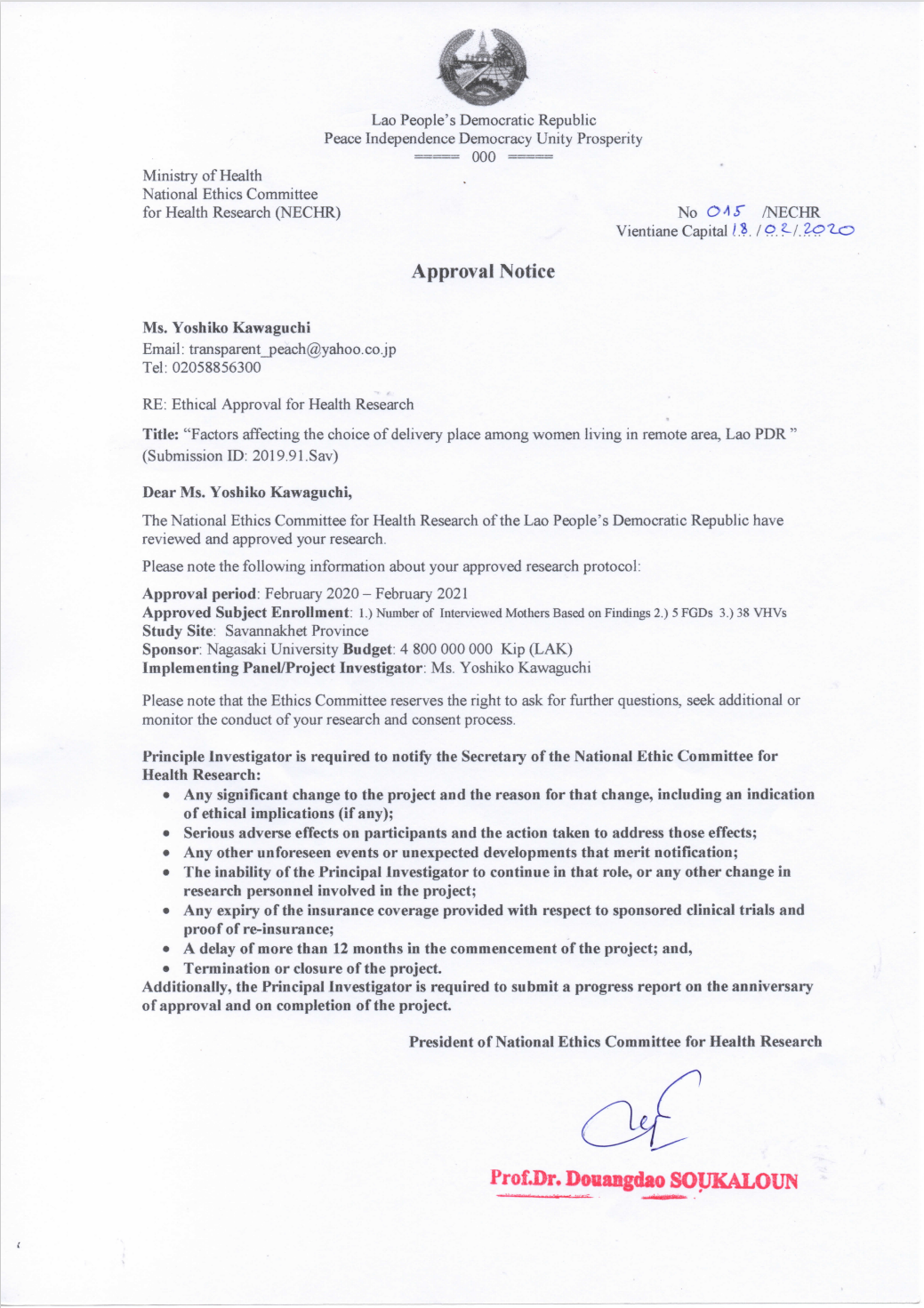


**Supplementary H: Women’s delivery history**

All delivery history is compared between HD and FD groups. It includes the latest deliveries.

|  | | HD | FD |
| --- | --- | --- | --- |
| Number of women | Primipara | 1 | 3 |
|  | Multipara | 6 | 6 |
| Total number of pregnancies | | 36^1)^ | 28 |
| Total number of deliveries | | 34 | 28 |
| Delivery place | Home | 34 | 10 |
|  | DHP | 0 | 9 |
|  | HC | 0 | 9 |
| Number of children | Total | 34 | 29^2)^ |
|  | Living children | 28 | 24 |
|  | Dead children | 6 | 5^3)^ |
|  | Women with child death(s) experience | 4 | 2 |
|  | Age of child death | | |
|  | Stillbirth | 0 | 1 |
|  | 2 days | 1 | 0 |
|  | 2 months | 3 | 0 |
|  | 3 months | 1 | 0 |
|  | 13 months | 1 | 0 |
|  | Not sure (not neonatal death) | 0 | 4 |

1) It includes two miscarriage. 2) It includes one stillbirth, one twin. 3) All five children were born from the mother of the ninth-para.

**Note:**

**1. HD experience in FD groups:** There were six multiparas in the FD group. Two of them had the experience of HD in the past; one woman had eight times HD experience, and another woman two times HD and two times FD before their latest childbirths. The other four multiparas experienced only FD.

**2. Child deaths:** Totally, ten children were dead after living births (exclude stillbirth); four HD women and one FD woman had experienced child death(s). All ten dead children were all born at home. However, these childbirths were explained as no problem by women.

**3. Stillbirth:** It happened at 24 weeks of pregnancy.
